# Transcriptome profiling of *L. infantum*-infected human macrophages reveals sex-specific type I interferon induction

**DOI:** 10.1371/journal.ppat.1013427

**Published:** 2025-08-12

**Authors:** Annika Bea, Helena Fehling, Fabian Hausmann, Fahten Margot Habib, Melanie Lütkemeyer, Lara Buer, Carola Schäfer, Charlotte Sophie Hansen, Barbara Honecker, Stefan Bonn, Bianca Elisabeth Schneider, Joachim Clos, Hanna Lotter

**Affiliations:** 1 Department for Molecular Infection Immunology, Bernhard Nocht Institute for Tropical Medicine, Hamburg, Germany; 2 Department for Leishmania genetics, Bernhard Nocht Institute for Tropical Medicine, Hamburg, Germany; 3 Institute of Medical Systems Bioinformatics, Hamburg Center for Translational Immunology (HCTI), Centre for Biomedical AI (bAIome), Center for Molecular Neuroscience (ZMNH), University Medical Center Hamburg-Eppendorf, Hamburg, Germany; 4 Host determinants in lung infections, Research Center Borstel - Leibniz Lung Center, Borstel, Germany; 5 German Center for Child and Adolescent Health (DZKJ), partner site Hamburg, University Medical Center Hamburg-Eppendorf, Hamburg, Germany; University of Geneva Faculty of Medicine: Universite de Geneve Faculte de Medecine, SWITZERLAND

## Abstract

Sex-based differences in the immune system influence the clinical course of infectious diseases, including many parasitic infections. Field studies of human infections and controlled experimental rodent models have shown that certain clinical forms of leishmaniasis occur more frequently in males. *Leishmania* parasites infect and proliferate in innate immune cells, particularly macrophages, and modulate early immune responses that constrain their survival and replication. In this study, we used a high-throughput *in vitro* system to assess sex differences in human macrophage-specific immunity to *Leishmania (L.) infantum* infection. Quantification of infection showed significantly higher infection rates and parasite loads in macrophages derived from men compared to those from women up to 76 hours post-infection (hpi). Evaluation of the macrophage phenotype during *L. infantum* infection revealed only minor changes in the proportions of primarily proinflammatory M1-like macrophages, whereas a reduction in the anti-inflammatory M2-like phenotype was observed in both sexes. Cytokine profiling revealed elevated levels of TNF, IL-8, IL-10, and reduced levels of IL-18 and CCL2 in culture supernatants over the time of infection. Transcriptomic analysis showed the highest adaptation of gene expression at 6 hpi, which was more pronounced in female-derived macrophages (1428 down-regulated/2145 up-regulated genes) compared to male-derived macrophages (972 down-regulated/1637 up-regulated genes), and gradually decreased over time in both sexes. Genes associated with type I interferon responses (e.g., *IFIT2, IFIT3, IFIT5, OASL, JAK1*), specific cytokine response (*IL-15, IL-1R1*), and the matrix metalloproteinase *MMP9* were up-regulated in female macrophages, while genes encoding proinflammatory chemokines involved in immune cell recruitment (*CXCL1, CXCL3, CCL20, CCL7*) were up-regulated in male macrophages. Treatment of infected macrophages with estradiol conferred marginal resistance to infection in female-derived macrophages, whereas testosterone treatment had no effect. In summary, our findings reveal immune mediators and underscore a biological sex difference that may explain females’ superior ability to combat *Leishmania* infections.

## Author summary

Women generally mount stronger immune responses than men, offering better protection against infectious diseases, including leishmaniasis - a vector-borne parasitic disease. Depending on the infecting *Leishmania* species, the disease causes various clinical forms. Across all manifestations, leishmaniasis tends to impact men more severely, as shown by epidemiological studies and reproduced in experimental models of the disease. In our study, we analyzed the effects of *Leishmania infantum* infection on isolated human macrophages from men and women, as macrophages act as the main mammalian host cell. Through high-throughput screening, we found that macrophages from male donors showed higher infection rates and parasite loads than those from female donors, even in *in vitro* monocultures. While macrophage polarization and cytokine release were similar between sexes, our transcriptomic analysis indicates a protective type I interferon response in female macrophages. In contrast, male macrophages exhibited increased expression of chemokines, potentially recruiting more immune cells. Moreover, estrogen treatment slightly boosted resistance to *Leishmania* in female macrophages, while testosterone exhibited no effect. These findings confirm a stronger anti-parasitic response in women, adding to our understanding of the biological determinants for their increased resilience to leishmaniasis, which could be crucial for developing individualized treatment regimens.

## Introduction

Many parasitic diseases show a male-biased incidence, morbidity, and mortality. This results from various factors, including sociocultural aspects, such as increased vector exposure in male-dominated work settings, and biological factors, such as chromosomal and hormonal influences. Biological factors especially affect immune protection, with females generally mounting more effective responses during infections, providing an advantage under infection pressure [1,2].

Leishmaniasis, which is still one of the most important neglected tropical diseases, is an example of this male-biased susceptibility. Depending on the infecting *Leishmania* species and host immune status, infections can manifest as cutaneous leishmaniasis (CL) characterized by self-healing skin ulcers, up to a disseminated, visceral infection (VL). After transmission by female *Phlebotomus* sandflies, *Leishmania* parasites primarily infect and proliferate in resident and recruited innate immune cells within the skin. Consequently, sex-based differences in host immune mechanisms may influence the ability of the parasite to evade clearance [3].

Macrophages are the primary host cells for *Leishmania* and exhibit remarkable plasticity. They respond to external stimuli by polarizing into either classically activated (M1), pro-inflammatory macrophages or alternatively activated (M2), anti-inflammatory macrophages [4]. M1 macrophages promote parasite elimination through the production of pro-inflammatory cytokines, whereas M2 macrophages support parasite persistence by secreting anti-inflammatory cytokines and arginase. Importantly, while M1 responses help control infection, excessive inflammation can cause tissue damage. Conversely, M2 macrophages contribute to wound healing and are particularly important in later stages of disease [5–8].

Epidemiological studies on the subject, which controlled for cultural and behavioral factors, still report sex differences in disease outcomes. Male predominance was observed in VL, both in New and Old World settings [9–11], as well as in CL caused by infection with *L. panamensis, L. braziliensis* or *L. mexicana* in the New World. The discrepancy between the sexes in the latter infection was associated with elevated serum levels of GM-CSF and IFN-γ in females [12–14]. Moreover, differences in tropism and disease manifestation between sexes were observed in *L. tropica* infection, which manifested more commonly as CL in females and as VL in males. Similarly, in *L. mexicana* infections, higher case numbers of anergic diffuse CL (ADCL) were observed in males, whereas localized CL was more prominent in females, potentially caused by an increased number and activity of Natural Killer cells and resulting increased levels of tumor necrosis factor (TNF) and interferon γ (IFN-γ). Notably, the strongest sex differences are observed in adults aged 20–59, suggesting a role for sex hormones in modulating susceptibility [15–19].

Sex-based differences in Leishmania infections are also evident in animal models [20]. In rodent models of *Leishmania* infection, males show increased susceptibility to infection, characterized by larger lesions and higher levels of Th2-associated cytokines such as IL-4, IL-10, and TGF-β, along with increased eosinophil recruitment [18,21,22]. In contrast, female animals showed smaller lesions and lower parasite burdens correlated with increased levels of pro-inflammatory mediators, including nitric oxide (NO), IL-12, IL-6, IL-1-β and IFN-γ [18,21,23]. Additionally, *in vivo* and *in vitro* experiments highlighted the significant influence of sex hormones in the outcome of CL and VL, with gonadectomized male mice exhibiting lower liver parasite burdens, and testosterone supplementation in female mice and hamsters resulting in increased *Leishmania* loads [21,24,25]. *In vitro,* murine estradiol-treated macrophages exhibit enhanced parasite killing [23,26], whereas testosterone promoted lower antimicrobial activities and increased parasite uptake [27,28].

By using a high content screening approach, flow cytometry and RNA sequencing, we studied sex differences in human macrophages infected with *L. infantum*. Higher infection rates and parasite loads were detected in macrophages from men, especially in later phases of infection. Interestingly, *L. infantum* infection reduced the M2 polarization of macrophages in both sexes, although the effect was more pronounced in macrophages from females. Cytokine analysis revealed a time-dependent increase in TNF, IL-8, and IL-10, with only minimal differences between sexes. The strongest gene expression changes occurred early post-infection, particularly in macrophages from women. Both sexes showed activation of inflammatory pathways, while female-specific differentially expressed genes (DEGs) were linked to protective interferon signaling pathways. Genes encoding specific chemokines, such as C-X-C motif chemokine (CXCL)-1, among others, were specifically up-regulated in macrophages from men. Estradiol treatment of infected macrophages resulted in a slight reduction in the parasite burden, but only in those derived from females, while testosterone treatment had no effect on infection in this system.

The results obtained in our experimental leishmaniasis model support the sex-specific paradigm with stronger protective, interferon-mediated immune response in macrophages from females, suggesting molecular markers for their greater resistance to *Leishmania* infection.

## Results

### Mature macrophages from male donors exhibit higher infection rates and parasite loads

Human epidemiological studies, animal models and *in vitro* studies using murine bone-marrow-derived macrophages have documented sex-based differences in the infection rates of *Leishmania* parasites and severity of leishmaniasis [18,20].

**Fig 1:**
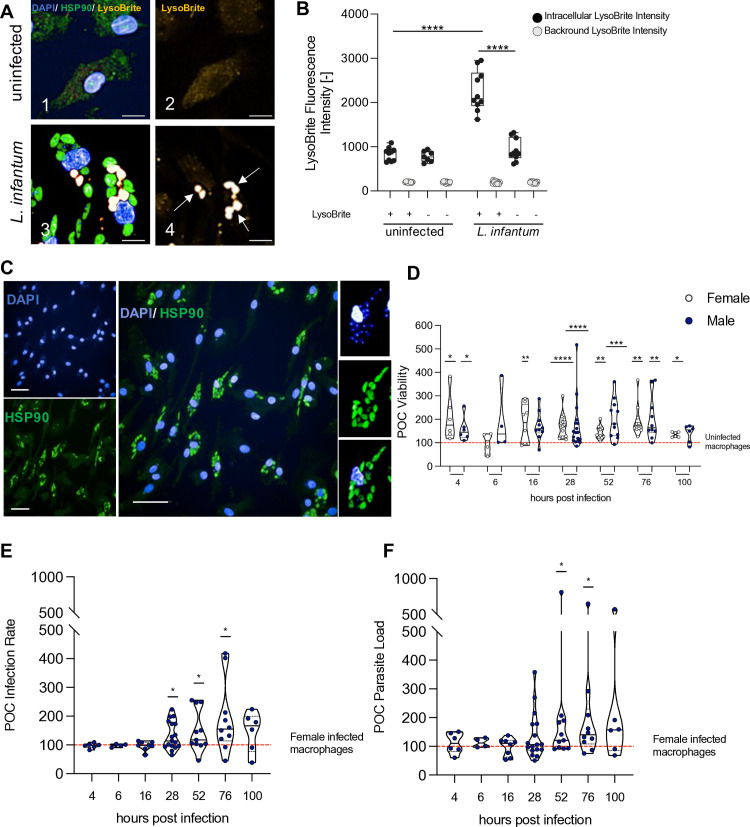
Intracellular localization of *Leishmania infantum* and sex-specific differences in human macrophage infection.

To assess the sex bias in the *in vitro* infection of primary human macrophages and its implications for disease progression, we investigated the course of *L. infantum* infection in macrophages derived from monocytes of both female and male donors. Parasite internalization *in vitro* was confirmed by the identification of the parasitophorous vacuole (PV), an intracellular compartment established by the parasites to facilitate their differentiation and replication. PVs partly consist of endolysosomal components of the host and therefore exhibit an acidic pH [29]. Using a lysosomotropic fluorescent dye (LysoBrite Red DND-99), PVs can be labelled and intracellular localization of the parasites can be determined (Fig 1A and 1B). Upon infection with any *Leishmania infantum*, a clear induction of the LysoBrite Red DND-99 signal was observed. The signals are seen in clear circular structures of 3–5 µm diameter around the early *L. infantum* amastigotes (Fig 1A). When quantifying the fluorescence intensity of the LysoBrite Red DND-99 signal within macrophages, significantly higher intensities were observed in infected macrophages compared to uninfected cells and cells without LysoBrite Red DND-99 staining (Fig 1B).

Fluorescent staining with 4,6-Diamidin-2-phenylindole (DAPI) and a parasite-specific, pan-*Leishmania* heat shock protein specific (α-LHSP90) antibody was used to identify intracellular parasites and monitored by high-content, image-based quantification at various time points post-infection (Fig 1C). The viability of human monocyte-derived macrophages (hMDMs), defined as the total number of cells per well, was the first investigated parameter. With the exception of 6 hpi for both sexes and 16 and 100 hpi for male macrophages, a significant increase in the viability of approximately 60% was observed in infected cells compared to uninfected macrophages. However, the viability of infected cells did not differ significantly between sexes (Fig 1D). Macrophages demonstrated a high initial infection rate of 90% of cells at 4 hpi, which significantly declined from 28 hpi onwards in both sexes to approximately 30% at 100 hpi (S1A Fig). When comparing the median percentage of infected cells between sexes at different time points after infection, the most pronounced difference was observed at 28 hpi (♀ = 38.9%, ♂ = 49.5%), although not significant due to the high variance. In accordance with these unnormalized data, direct comparison of the percentages of infected macrophages between both sexes by normalization of male-derived infected macrophages to female-derived macrophages, revealed no difference at initial time points and the last time point evaluated. However, a significantly increased infection rate was observed in male-derived cells compared to female-derived cells from 28 to 76 hpi, reaching peak differences at 76 hpi (♂ = 155%) (Fig 1E). Infected cells from female and male donors contained a median of 8–9 parasites at the time extracellular parasites were removed (4 hpi), but the number significantly declined from 28 hpi onwards until only 2 parasites were detected at 100 hpi, with no significant differences between the sexes (S1B Fig). Normalization of the parasite load to values of female-derived cells showed a clear increase of the number of intracellular parasites in male-derived macrophages from 52 hpi to 100 hpi, which was found to be significant at 52 and 76 hpi (52 hpi: ♂ = 120,7%; 76 hpi: ♂ = 140%) (Fig 1F).

In conclusion, infection with *L. infantum* led to increased viability of macrophages in both sexes. However, after normalization to account for inter-experimental variability, male-derived macrophages exhibited higher infection rates and parasite loads compared to their female-derived counterparts.

Mature macrophages derived from female and male human blood donors were infected with *L. infantum* metacyclic promastigotes (MOI 15:1). Infection parameters were quantified using the Opera Phenix confocal microscope and a customized image analysis pipeline (see S1 Table). To assess the intracellular localization of parasites, cells were incubated with LysoBrite Red DND-99 for 2 hours at 37 °C and 5% CO₂ prior to fixation. (A) Representative images showing parasitophorous vacuoles (PVs) within human macrophages 24 hours post-infection (hpi). Nuclei were stained with DAPI (blue), *Leishmania* parasites with α-LHSP90-AF488 (green), and PVs with LysoBrite detected in the AF568 channel (yellow). 1–2: uninfected macrophages; 3–4: infected macrophages. Arrows indicate intracellular PVs. Scale bar = 50 μm. (B) Quantification of PV fluorescence intensity using Harmony image analysis software. Data are presented as a box plot (n = 10 independent PVs per infected cell). Statistical analysis: one-way ANOVA with Dunnett’s correction (vs. uninfected control) and two-tailed paired Student’s t-test (vs. control without LysoBrite). (C) Representative microscopy images of infected macrophages stained with DAPI and α-LHSP90-AF488. Scale bars: 100 μm (overview), 20 μm (close-up). (D) Viability of infected cells normalized to uninfected controls (red dotted line), shown separately for each sex. (E) Infection rate and (F) parasite load of male-derived macrophages normalized to the mean of infected female-derived macrophages (red dotted line). Data are displayed as violin plots.

n_F/M 4hpi_ = 6/6, n_F/M 6hpi_ = 4/4, n_F/M 16hpi_ = 10/8, n_F/M 28hpi_ = 19/17, n_F/M 52hpi_ = 11/11, n_F/M 76hpi_ = 10/10, n_F/M 100hpi_ = 6/6. P-values were calculated using One sample t-test compared to respective controls (*p < 0.05, **p < 0.01, ***p < 0.001, ****p < 0.0001). POC = Percentage of Control

### *L. infantum* infection skews macrophage polarization towards less M2-like macrophages, which is more pronounced in female cells

The process of macrophage polarization is crucial for the control and immune response to leishmaniasis [5,6,8]. Since previous reports showed that protective, pro-inflammatory M1- and permissive, anti-inflammatory M2-like human and mouse macrophages have distinct morphologies [30], we developed a high-throughput phenotypic screen to analyze a potential sex-specific impact on polarization during *Leishmania* infection in hMDMs *in vitro*.

**Fig 2:**
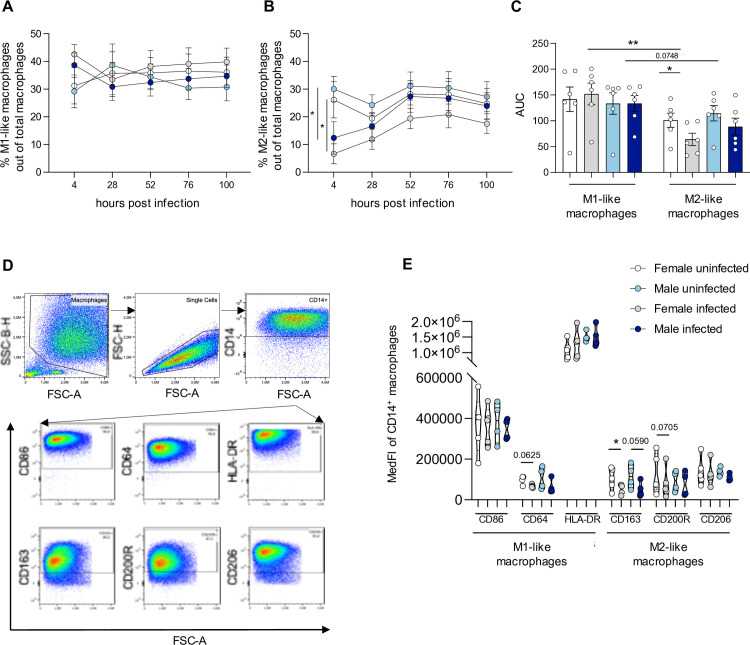
*L. infantum* infection induces changes in M1/M2 polarization of human macrophages.

Based on the correlation between cell shape and macrophage activation we designed an image analysis sequence (using Harmony software) to either detect M1-like macrophages, which exhibit a homogenous, roundish cell morphology or M2-like macrophages, which present as spindle-shaped elongated cells with cytoplasmic extensions [31]. A detailed validation of the phenotypic screening method is shown in S2 Fig. On this basis, *Leishmania*-infected male and female derived macrophages were examined for their polarization state over the course of the infection. In comparison to uninfected cells (~ 35% M1-like macrophages), *Leishmania*-infected macrophages showed a slight increase in M1-like macrophage in both sexes at 4 hpi (♀ = 42.5%, ♂ = 38.6%) (Fig 2A). For M2-like macrophages, a mean proportion of 20–30% was observed in uninfected cells throughout the cultivation time period. Upon infection, a significant reduction of M2-like macrophages was observed at 4 hpi (♀ = 6.6%, ♂ = 12.5%) followed by a gradual increase as the infection progressed (Fig 2B). Analysis of the area under the curve (AUC) revealed a significantly higher proportion of pro-inflammatory M1-like macrophages in female-derived cells following *Leishmania* infection, compared to anti-inflammatory M2-like macrophages. M1-like macrophages are typically associated with intracellular parasite control and host protection, whereas M2-like macrophages have been linked to disease progression [5]. In addition, a significant reduction in M2-like macrophages was observed in infected female-derived cells relative to uninfected controls. In contrast, *Leishmania*-infected macrophages from male donors did not display significant differences in the proportions of M1-like and M2-like subsets; however, a non-significant trend toward reduced M2-like macrophage numbers was noted during infection (Fig 2C).

These results were supported when analyzing the expression M1- and M2-related surface markers on infected Cluster of differentiation (CD) 14 positive macrophages at 28 hpi, examining the influence of intracellular amastigotes on macrophage polarization. The analyses were performed following the established gating strategy (Fig 2D). No significant differences in the Median Fluorescent Intensity (MedFI) of M1-related markers were observed on infected macrophages of both sexes compared to respective naïve controls, only a significant decrease in the MedFI of CD163 (M2-related marker) was measured for infected female-derived samples compared to uninfected samples. No significant differences were found for the M2-related markers CD200R and CD206 (Fig 2E).

Overall, these results show that infection with *L. infantum* leads to a shift in macrophage polarization, with a slight increase in M1-like macrophages and a decrease in M2-like macrophages in both sexes. However, phenotypic screening and validation by flow cytometry showed that the decrease on M2-like macrophages tends to be more pronounced in female-derived macrophages.

Macrophages from female and male blood donors were infected with *L. infantum* metacyclic promastigotes (MOI of 15:1) and incubated for the indicated time periods. Depicted are the proportion of (A) M1-like, and (B) M2-like uninfected and infected macrophages as determined by an established morphology-based image analysis sequence (see. S2 Fig and S1 Table) using Harmony software (C). Display of the area under the curve (AUC) of graphs A and B. Data represent mean ± SEM. n_F/M_ = 6/6 (D) Representative gating strategy used to decipher M1-and M2 polarization of CD14^+^ macrophages as determined by flow cytometry (M1 markers: CD86, CD64, HLA-DR; M2 markers: CD163, CD200R, CD206). (E) Box plot of MedFI of M1- and M2-related surface markers on naïve macrophages at 28 hpi. n_F/M_ = 5/4. P-values were calculated (A + B) using two-way ANOVA analysis and uncorrected Fisher´s LSD, (C) two-tailed paired Student ´s t- test for comparison of different infection conditions within one sex or two-tailed unpaired Student ´s t-test, (D) two-tailed paired analysis Student´s *t*-test (*p < 0.05, **p < 0.01).

### *L. infantum* infection of macrophages induces a distinct release of pro- and anti-inflammatory cytokines

**Fig 3:**
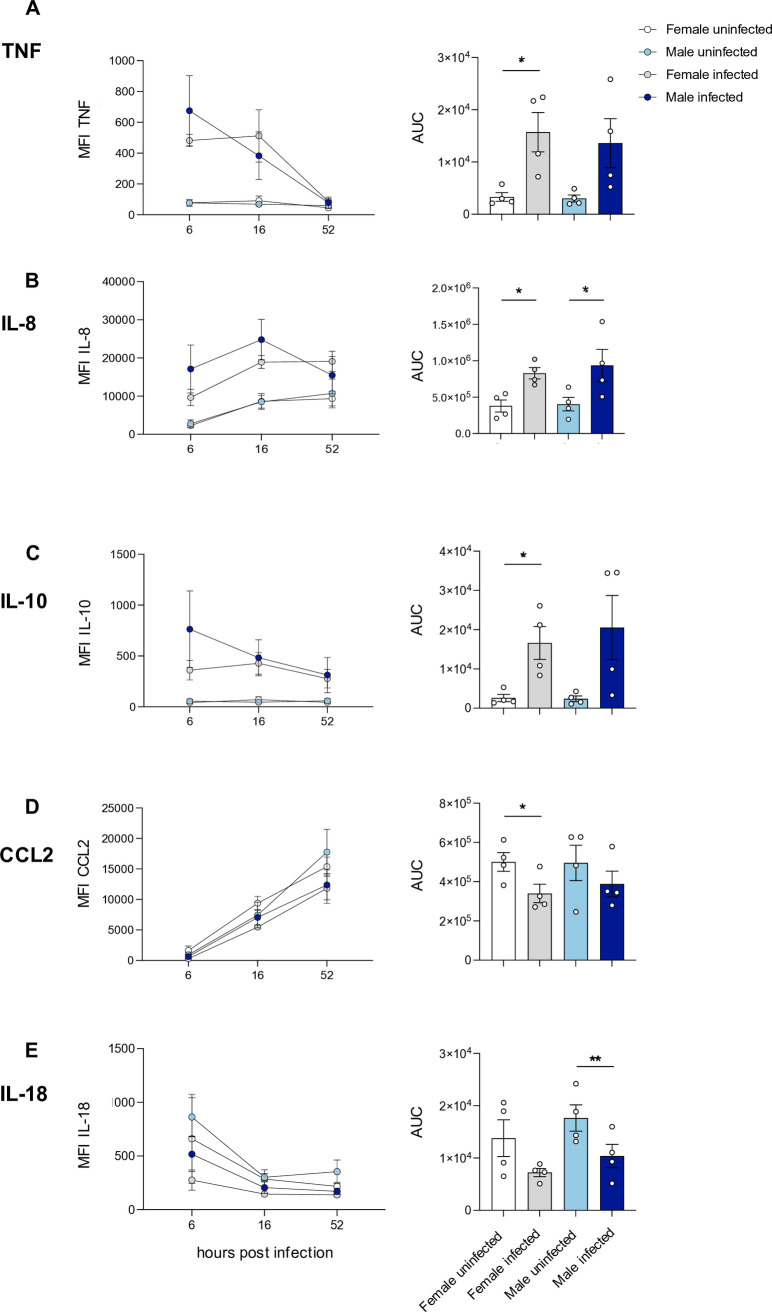
Infection-specific cytokines produced during *L. infantum*-infection in macrophages derived from men and women. Mature macrophages from female and male donors were infected with *L. infantum (*MOI 15:1) for 6 to 52 hours. Cytokines were determined in the culture supernatant using cytometric bead assay (LEGENDplex). Time-course analysis of the MFI of (A) TNF (B) IL-8 (C) IL-10 (D) CCL2 and (E) IL-18 at indicated time points (left side) and respective area under the curve (AUC) (right side). Data represent mean ± SEM, n_F/M _= 4/4. P-values were calculated using two-tailed paired analysis Student´s *t*-test (*p < 0.05, **p < 0.01).

Based on the previous experiments, we analyzed the cytokine profile in the supernatant of *L. infantum* infected macrophages from men and women at an early time point to monitor the effect of the initial contact between parasites and macrophages (6 hpi), a time point to better unravel the first significant differences in infection rates seen in previous experiments (16 hpi) and a time point in the later infection where the differences are solidified (52 hpi). We measured a panel of well-characterized pro-inflammatory cytokines and chemokines known to be secreted by macrophages in response to infection [32,33]. In the context of *L. infantum*-induced visceral leishmaniasis, we assessed immune mediators including TNF, IL-1β, CXCL10, IL-12p70, IL-18, IL-23, and C-C motif chemokine ligand 2 (CCL2). To evaluate potential susceptibility factors, we also included anti-inflammatory markers such as IL-10 and Arginase. All analytes were quantified using a bead-based multiplex assay (LEGENDplex). A pronounced, infection-dependent increase in the expression levels of TNF, IL-8, and IL-10 was observed over the course of infection, with the most substantial upregulation occurring at the earliest time point (Fig 3A–C). Quantitative analysis based on the area under the curve (AUC) confirmed a significant induction of all three cytokines in supernatants of female-derived macrophages, and a significant increase in IL-8 in male-derived macrophages (Fig 3A–C). In the case of CCL2, expression levels increased progressively over time in both infected and uninfected cultures; however, concentrations remained consistently higher in the uninfected controls (Fig 3D). Conversely, IL-18 levels - which were initially elevated in uninfected macrophages - declined markedly following infection, with a statistically significant reduction observed particularly in samples from male-derived macrophages (Fig 3E).

For the other measured cytokines, IL-1, CXCL10, IL-23, Arginase, CCL17 and IL-12p70 no significant infection- or sex-dependent differences were observed (S3A-S3F Fig).

In summary, TNF, IL-8, and IL-10 specifically increased following infection, whereas CCL2 and IL-18 showed an infection-dependent reduction. Overall, only slight sex differences were observed in the cytokine profile of *L. infantum*-infected macrophages.

### *L. infantum* infection induces most pronounced changes in early gene expression

**Fig 4:**
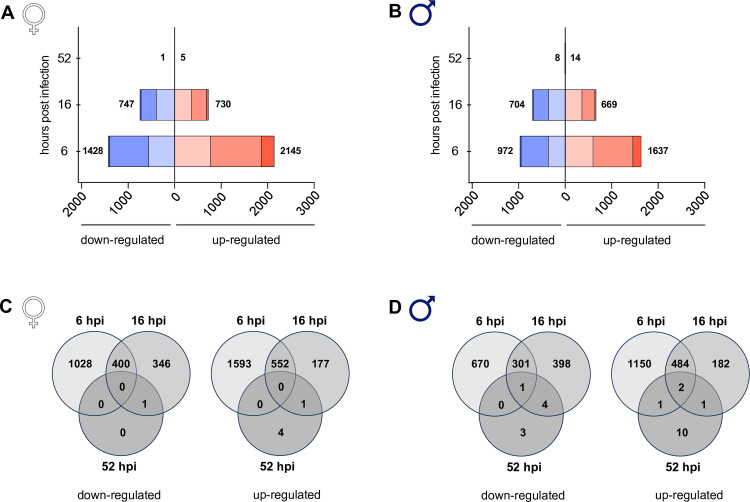
Analysis of differentially expressed genes between uninfected and infected macrophages from female and male donors. Mature macrophages from women and men were infected with *L. infantum* (MOI 15:1). mRNA from infected macrophages and naïve controls was isolated and subjected to whole transcriptome sequencing. Number of differentially expressed genes (adj. *p* < 0.05) at different time points after *L. infantum* infection in relation to uninfected controls for macrophages from (A) women and (B) men. Box length depicts number of differently expressed genes down-regulated (left, blue) and up-regulated (right, red). Color shading correlates with the magnitude of regulation (dark: log_2_(fold change)>3; medium: 1 < log_2_(fold change) <3, light: log_2_(fold change) <1). (C, D) Venn diagrams depicting differentially regulated genes (adj. *p* < 0.05) at different time points after *L. infantum* infection of macrophages from (C) women and (D) men compared to uninfected controls. n_F/M_ = 4/4.

We next conducted RNA sequencing of infected macrophages from female and male donors to test for potential sex-specific differences at the mRNA level. When comparing *L. infantum*-infected macrophages from both sexes with corresponding uninfected controls, the highest number of significantly differentially expressed genes (DEGs) (adj. p < 0.05) was observed at the earliest time point, 6 hpi, with a higher number of DEGs observed in female-derived macrophages (♀ = 3573, ♂ = 2609). In both sexes, more genes were up-regulated (♀ = 2145/3573, ♂ = 1637/2609) than down-regulated (♀ = 1428/3573, ♂ = 972/2609). Less DEGs were observed for either sex at 16 hpi compared to 6 hpi, with comparable numbers of DEGs for female and male macrophages (♀ = 1477, ♂ = 1373) and similar proportions of up- (♀ = 730/1477, ♂ = 669/1373) and down-regulated (♀ = 747/1477, ♂ = 704/1373) genes. Lastly, almost no DEGs were observed at 52 hpi (♀ =  6, ♂ =  22) (Fig 4A and 4B and S2 and S3 Tables).

This result was mirrored when Principal Component Analysis (PCA) was applied to each time point and sex separately. This revealed two distinct clusters for both sexes at 6 and 16 hpi, which can be allocated to uninfected (grey) and infected (green) samples, whereas a clear clustering of donor pairs was observed at 52 hpi, indicated by node shape (S4A and S4B Fig). Comparing the DEGs within each sex at different time points, most of the genes were exclusively differentially expressed at 6 hpi. However, there was a notable overlap of about 30% of the genes that were also observed at 16 hpi, corresponding to a proportion of approximately 40–50% of the down-regulated genes and over 70% of the up-regulated genes at 16 hpi. In female samples, no gene was found to be regulated at all investigated time points, whereas one down-regulated gene (*Zinc finger protein 704* (*ZNF704*)) and two up-regulated genes (*EMILIN1, IL7R*) were found over the total course of infection in male samples (Fig 4C and 4D and S2 and S3 Tables).

Our findings show an early gene expression remodeling in human macrophages upon infection with *L. infantum* in both sexes that gradually diminished over time. Notably, there was a distinct overlap in the DEGs across the time points, with a stronger adaptation of the gene expression in female macrophages at early time points of infection.

### *L. infantum* infected macrophages exhibit a pro-inflammatory gene expression profile

**Fig 5:**
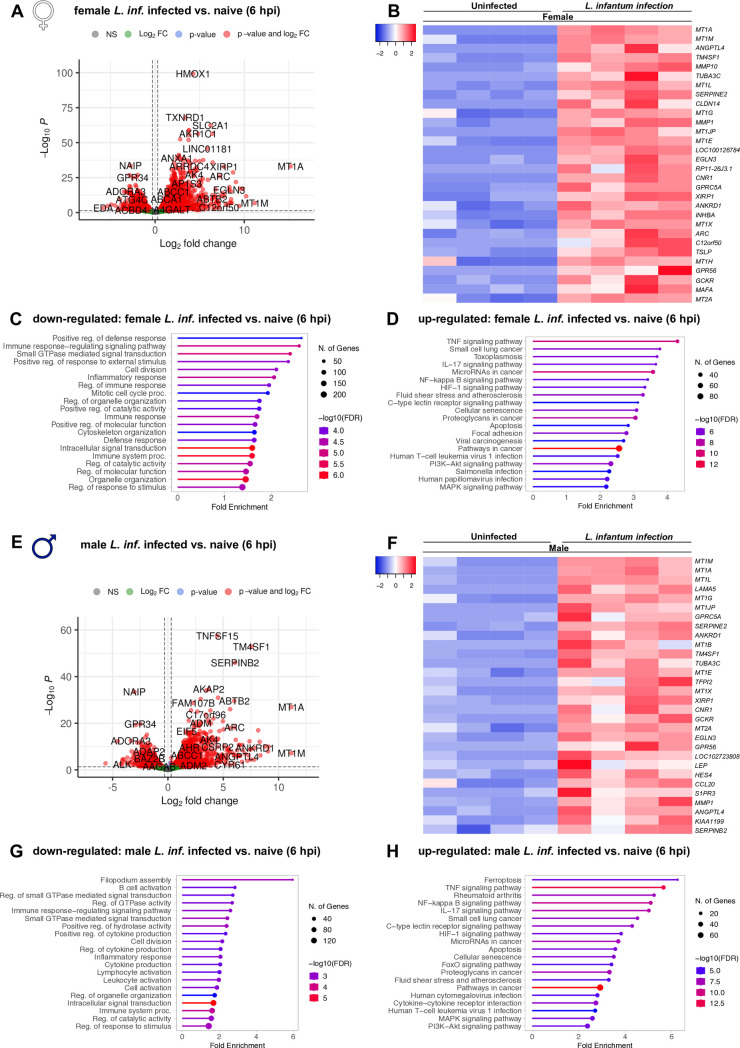
Transcriptional analysis of *L. infantum*-infected macrophages from female and male donors 6 hpi. Mature macrophages from (A-D) women and (E-H) men were infected with *L. infantum* (MOI 15:1) for 6 hours. mRNA from infected macrophages and naïve controls was isolated and subjected to whole transcriptome sequencing. (A, E) Volcano plots depicting differential gene expression between uninfected and infected macrophages according to magnitude of change (Log2(fold change)) and statistical significance of change (-Log10(adjusted p-value). Genes significantly regulated between the conditions (Log2(fold change) > I0.25I, adj. p < 0.05) are marked in red (left side = low in infection, right side = high in infection). (B, F) Heatmaps depict top 30 regulated genes. (D, H) KEGG pathway analysis of significantly up-regulated genes in infection (adj. p < 0.05, log2 (fold change) >1) and (C, G) Biological processes GO term enrichment of significantly down-regulated genes in infection (adj. p < 0.05, log2 (fold change) <-1). (C, D, G, H) Shown are the top 20 pathways/processes sorted by fold enrichment. Significance of enrichment indicated by color and number of genes in pathway indicated in node size. n_F/M_ = 4/4.

Analyzing the DEGs (Log_2_(fold change) > I1I, adj. p < 0.05), many members of the metallothionein family 1 (MT1) were found among the top differentially expressed genes at 6 and 16 hpi in both sexes (Figs 5A 5B, 5E, 5F,S5A, S5B, S5E and S5F). Kyoto Encyclopedia of Genes and Genomes (KEGG) pathway analysis of genes up-regulated between uninfected and *L. infantum*-infected macrophages was performed to identify activated cellular pathways in response to infection at different time points. The top up-regulated pathways detected at 6 hpi were similar in both sexes and included mainly immunity-related pathways such as TNF signaling, NF-B signaling pathway, hypoxia- inducible factor 1 (HIF-1) signaling pathway and IL-17 signaling (Fig 5D and 5H). Genes up-regulated at 16 hpi partly allocated to previously described immune pathways in both sexes resulting in their identification among the top- regulated pathways again (e.g., TNF signaling and peroxisome proliferator-activated receptor (PPAR) signaling), although less prominently enriched. In addition, similar metabolic pathways (mineral absorption, chemical carcinogenesis-reactive oxygen species) or individual metabolic pathways, such as steroid hormone biosynthesis and cholesterol metabolism in female samples and glutathione metabolism in male samples were affected at 16 hpi (S5D and S5H Fig). Corresponding Biological processes GO term enrichment analyses are listed in S6B, S6D, S6F and S6H Fig.

Biological processes Gene ontology (GO) term enrichment was performed to determine involved processes allocated to the down-regulated genes between infected compared with uninfected macrophages of the same sex. At 6 hpi, most of the down-regulated DEGs were involved in the immune response as well, resulting in an enrichment of immune response-related pathways. For female-derived samples, down-regulated DEGs were categorized in processes such as inflammatory response, innate immune response, regulation of defense response and cytokine production (Fig 5C), whereas male-derived samples showed reduced expression of genes associated with macrophage migration, regulation of inflammatory response and B-cell activation, to name the important ones (Fig 5G). For male-derived samples, the downregulation of genes allocated to B-cell activity became even more apparent at 16 hpi, at which many DEGs were defined as part of antibody-dependent cellular cytotoxicity or B-cell receptor signaling process. All other affected processes were also connected to immunity and included processes such as cytokine production or regulation of leukocyte proliferation (S5G Fig). For female samples, most down-regulated processes at 16 hpi were part of the cellular response to stimuli (cellular response to reactive oxygen species or oxidative stress), immune response (inflammatory response, regulation of immune system production) or cytokine production (S5C Fig). No KEGG pathway or GO term enrichment analysis could be performed for the last time point due to low number of DEGs (Fig 4A and 4B). Corresponding KEGG pathway analyses are listed in S6A, S6C, S6Eand S6G Fig.

Taken together, these results show the induction of a predominantly pro-inflammatory response to *L. infantum* infection, which is indicated by the regulation of key immune-related pathways in both sexes on mRNA level.

### *L. infantum* infection induces a sex-specific activation of immune-relevant pathways

**Fig 6:**
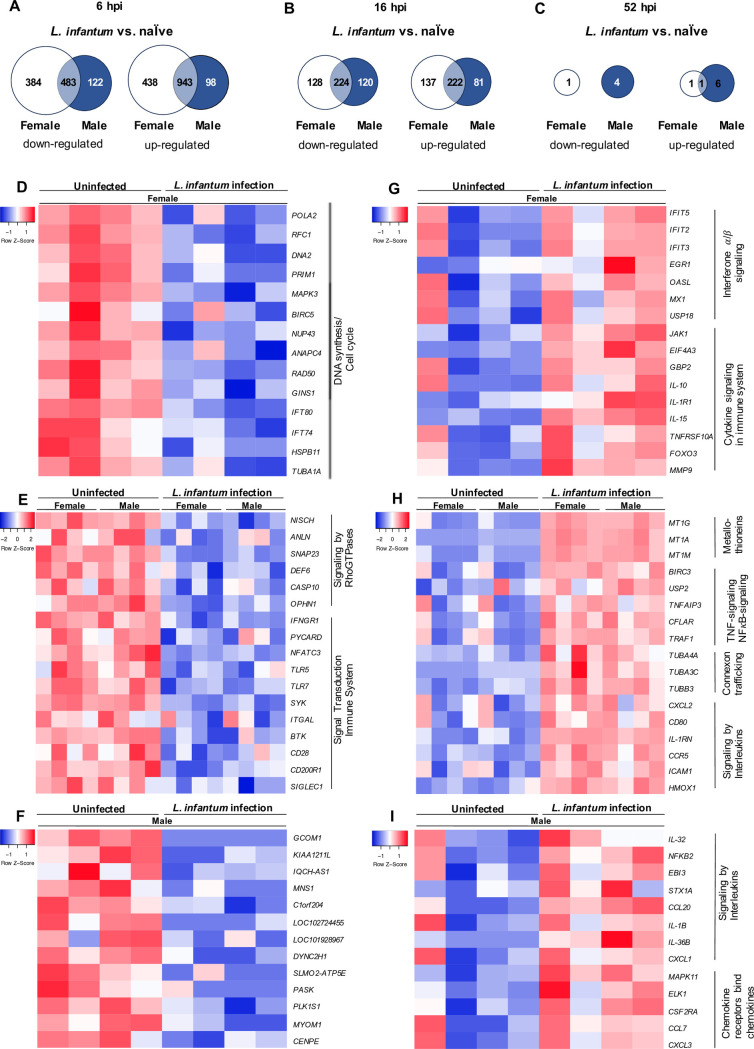
Sex differences in the transcriptome of *L. infantum*-infected macrophages. Mature macrophages from female and male donors were infected with *L. infantum* (MOI 15:1) for 6 hours. mRNA from infected macrophages and naïve controls was isolated and subjected to whole transcriptome sequencing. **(A-C)** Venn diagrams depicting the DEGs between female and male donors following *L. infantum* infection compared to corresponding naïve control (adj. *p* < 0.05, log2 (fold change) <|1|) at (A) 6, (B) 16 and (C) 52 hpi. For 6 hpi (D-I) top enriched pathways identified using Reactome database are depicted at the side of the heatmap. Heatmaps depict selected corresponding genes for (D) exclusively down-regulated genes in female samples, (E) similarly down-regulated genes in both female and male samples and (F) exclusively down-regulated genes in male samples, (G) exclusively up-regulated genes in female samples, (H) similarly up-regulated genes in both female and male samples and (I) exclusively up-regulated genes in male samples,.

To further investigate the sex bias in gene regulation upon infection, DEGs of uninfected and *L. infantum-*infected macrophages of male and female origin were compared to find regulated genes that were specific for each sex (Log_2_(fold change) > I1I, adj. p < 0.05). The results were depicted as a Venn diagram separately for up- and down-regulated genes and for the different investigation time points. This analysis showed that, except for 52 hpi, the largest proportion of about 60–90% of detected genes were similarly up- and down-regulated in both sexes (Fig 6A-C). Each subset of similarly or exclusively expressed genes was used to determine characteristic pathways affected using the Reactome database (https://reactome.org/). The top regulated pathways were depicted in a heat map with selected associated genes.

Analyzing the up-regulated genes in a sex-dependent manner, many genes involved in the interferon α/β signaling pathway could be exclusively found in female macrophages at 6 hpi. Among others, *2’-5’ oligoadenylate synthetase-like (OASL), Interferon induced protein with tetratricopeptide repeats* 2, *3* and *5* (*IFIT2, IFIT3, IFIT5*) were up-regulated in female-derived macrophages upon infection. In addition, cytokine-encoding genes, such as *IL15 and IL10* were also exclusively up-regulated in female samples at the initial time point (Fig 6D). At 16 hpi, genes which were solely differentially expressed in infected female macrophages play a role in NFB signaling, such as *Tumor necrosis factor ligand superfamily member 14* (*TNFSF14*)*, Tumor necrosis factor receptor superfamily member 12A* (*TNFRSF12A*) *and CD40* (S7A Fig). Exclusively down-regulated genes in female macrophages at 6 hpi were mostly categorized in pathways of DNA replication and cell cycle, with genes such as *DNA polymerase subunit 2* (*POLA2*), *DNA replication ATP-dependent helicase/nuclease DNA2 (DNA2)* and *respiratory chain factor subunit 1 (RFC1)* (Fig 6E), whereas at 16 hpi, exclusively down-regulated genes in female infected cells were associated with cholesterol biosynthesis (S7B Fig).

In accordance with the previously performed analyses, genes similarly up-regulated in female-, and male-derived macrophages with the strongest transcriptional regulation at 6 and 16 hpi were genes encoding proteins of the MT1 family (e.g., *MT1H* and *MT1A*) that were associated with mineral absorption (Figs 6F and S7C). Additionally, many genes regulated in both sexes at 6 hpi were grouped into immunity-related pathways and cytokine responses or as part of connexon trafficking. Associated with these pathways were genes such as *heme oxygenase 1 (HMOX1)*, *CXCL2* and *CCL5* or genes of the tubulin family (Fig 6F). Interestingly, at 16 hpi, many genes coding for proteins of the matrix metalloproteases family (MMP) were found to be differentially expressed in both sexes and related to collagen degradation (S7C Fig). Analysis of the similarly down-regulated genes upon *L. infantum* infection between female and male macrophages revealed many genes coding for mediators of both innate and adaptive immune system at early time points (6 and 16 hpi). At both time points, genes encoding several surface markers (e.g., *CD200R1*, *CD163 and CD28*), partly associated with macrophage polarization, were differentially expressed (Figs 6Hand S7E).

Time-dependent decrease of the expression was observed for genes coding for Toll-like receptors (*TLR7*, *TLR5*), caspase 10 (*CASP10*) and Interferon gamma receptor 1 (*IFNGR1)* at 6 hpi (Fig 6G). At 16 hpi, lower expression of additional genes associated with *Leishmania* infection were observed in both sexes. Among others, these genes were Fc receptor*-*encoding genes (*FCGR1A, FCGR3A*) (S7D Fig). Lastly, exclusive expression of many genes encoding immune mediators, which allocate to signaling by interleukins or chemokines could be detected in male-derived macrophages upon infection at 6 hpi, such as *CXCL1*, *IL32*, *CCL7* and *IL1B (*Fig 6F). Clustering of DEGs associated to immune response, such as *Nacht, LRR and PYD domains-containing protein 1* (*NLRP1*) *and 3, FCGR2A and 2B,* were found when analyzing exclusive downregulation of genes in male samples at 16 hpi (S7F Fig). No significant enrichment could be performed for the exclusively differentially expressed genes in male samples at 6 hpi due to the small gene set (Fig 6I).

In summary, in spite of many similarly regulated host responses, our results show a clear sex-based distinction in the activation of immune-relevant pathways upon *L. infantum* infection, most prominently seen in the female-specific activation of interferon signaling.

### Stimulation with steroid hormones does not alter the infection rate or parasite burden of macrophages

**Fig 7:**
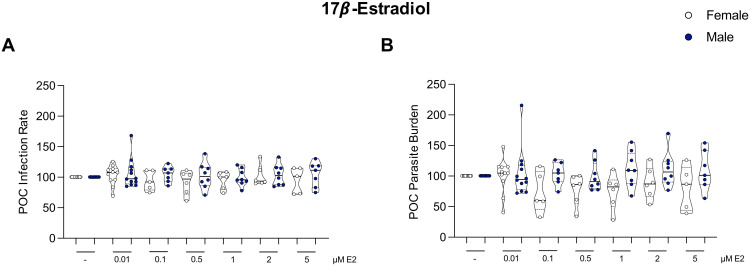
Influence of steroid hormone stimulation on *L. infantum* infection of macrophages. Mature macrophages from female and male donors were stimulated with 17-Estradiol (E2) in various concentrations (0.01– 5μM) before infection with *L. infantum* (MOI 15:1) for 28 hours. Parameters were quantified using Opera Phenix confocal microscope and customized image analysis sequence. **(A)** Infection rate and (B) parasite burden of macrophages normalized to corresponding infected unstimulated control (POC = 100). Data are shown as boxplot, E2 = n_F/M -_ = 12/13, n_F/M 0.01µM_ = 11/12, n_F/M 0.1µmM_ = 5/7, n_F/M 0.5µM_ = 6/8, n_F/M 1µM_ = 6/8, n_F/M 2µM_ = 6/8, n_F/M 5µM_ = 5/7.

Macrophages express both estrogen [34] and testosterone receptors [35,36] making them susceptible to immune regulation by steroid hormone binding, which has been shown to affect the outcome of *Leishmania* infections [37]. To investigate the putative influence of hormone stimulation on the *in vitro* infection with *L. infantum* parasites, mature macrophages from both female and male donors were treated with varying concentrations of 17-estradiol (E2) (Fig 7A and 7B) or 5-Dihydrotestosterone (DHT) (S8A and S8B Fig) before infection. Our preliminary results suggest that the infection rate of macrophages from both sexes remained unchanged when stimulated with either hormone compared to infected, untreated controls (Figs 7A and S8A). Interestingly, although no significant reduction in the overall parasite burden could be observed under E2 stimulation, a clear tendency of decreased parasite burden was observed in 40% of the female donors, whereas no difference was detected in male donors (Fig 7B) or in response to DHT stimulation (S8B Fig). No correlation was found between age of the donor and the reduction in parasite load.

To consolidate these results, the clearly limited sample sizes, especially for the DHT treatment, would need to be increased and different treatment regimens should be employed and compared.

## Discussion

Epidemiological studies have shown a male predominance of both CL and VL in endemic regions, with higher incidences of both forms and higher mortality for VL as summarized in the review by Lockard *et al.* [18]. This difference is most likely caused by a complex interaction of various determinants, such as infecting *Leishmania* spp., societal and environmental factors (host gender) as well as biological factors (host sex). Here, we wanted to determine the impact of host sex on the immune response to leishmaniasis by sex-specific characterization of human primary macrophages as the main host cells for *Leishmania* parasites, on the cellular, immunological and transcriptome level. This was partly done using the integration of automated multicolor fluorescence *in vitro* HCS assays based on the published image analysis sequence by Fehling *et al.* for murine macrophages [38], which was adapted for human primary macrophages, leading to the first report of sex-specific differences in an *in vitro* infection model based on primary human macrophages with *L. infantum*.

To date, evidence for sex differences in *Leishmania* infections has come primarily from experimental rodent models or epidemiological studies. The latter showed that male patients comprise the majority of clinical cases in VL, which was also reflected in animal models, where male mice and hamsters exhibited larger lesions and higher parasite loads upon infection with dermotropic *Leishmania* species [18,39]. By monitoring the infection rate and parasite load in macrophages derived from both female and male donors over time, significantly lower infection rates and fewer parasites per infected macrophage were noted in female-derived macrophages, starting at 28 hours following initial contact with the parasite. These observed differences allowed further characterization of infected macrophages to unravel underlying causes for the differing susceptibilities. However, limitations of the *in vitro* infection assay became apparent, as no parasite replication was observed and infection rates gradually decreased over time, not mimicking the natural situation entirely [40]. To a certain extent, the latter can probably be accounted for by optimizing the parasite preparation before infection [41]. This phenomenon has been observed by many other researchers and was associated with generally lower division rates of amastigotes, as well as with missing host factors in monocultures, that would be present in *in vitro* experiments involving multiple cell types or in *in vivo* experiments [40,42].

While macrophages are the primary host cells for *Leishmania*, neutrophils are the first immune cells recruited to the site of parasite inoculation. At this early stage, they play a crucial role in recognizing and eliminating the parasite and can influence the course of the infection directly, but also indirectly through their uptake by macrophages. Whether sex-specific differences exist in the neutrophil-mediated uptake of parasites - differences that could characterize the further infection dynamics - is still unknown. However, a corresponding *in vitro* approach to investigate sex-specific differences in the uptake of Leishmania parasites by neutrophils encounters several problems, although comparable *in vitro* assays exist [43]. These include their short lifespan of neutrophils [44], pronounced donor heterogeneity [45,46], and a low RNA content [47]. In addition, higher neutrophil counts in males [48] and conversely stronger innate immune response signatures in neutrophils of female individuals [49,50] complicate the analysis of such studies.

Independently of donor sex, *L. infantum* significantly prolonged viability and prevented apoptosis, as almost consistently more macrophages were present in infected samples than in uninfected samples. This has been shown for various *Leishmania* spp. with different underlying mechanisms, depending on species and *in vitro* system, summarized in a review from Solano-Gálvez *et al.* [51]. According to the authors, possible mechanism of pathogens inhibiting apoptosis to increase time for replication include modulation of host signaling pathways, expression of anti-apoptotic proteins or induction of host immune responses. The most common caspase-dependent pathway results in the activation of either caspase 8 or caspase 10, ultimately leading to the formation of apoptotic bodies [52]. Interestingly, in our experiments, caspase 10 showed significantly decreased expression on the RNA level in both sexes upon infection at 6 hpi. Caspase-dependent inhibition of apoptosis was also seen by *L. donovani* in bone marrow-derived macrophages and by *L. major* in neutrophils and hMDMs, respectively [53–55]. Additionally, increased expression of *PIK3CB* and *AKT3* (S2 and S3 Tables) as part of the PI3K/Akt signaling pathway in macrophages from either sex at 6 hpi can have regulatory effects on apoptosis, mainly by inactivation of pro-apoptotic proteins [56], described for other *Leishmania* spp. but not yet for *L. infantum* [54,57,58].

Utilizing the widely accepted morphology-based distinction between macrophage subsets [30,59], our study of the macrophage polarization state during *L. infantum* infection showed an initial decrease of the M2-like phenotype in all infected samples. However, female macrophages showed lower M2-like macrophage counts throughout the observation period, indicating reduced amounts of anti-inflammatory macrophages. Similar results were obtained on the marker protein level and in the transcriptome profiling, where significantly down-regulated M2-macrophage-related surface markers were found. Genes encoding CD200R1 and CD163, previously described markers for M2 polarization [60], were equally down-regulated in both sexes at 6 hpi and 16 hpi on mRNA and at 28 hpi on protein level, also showing a stronger tendency of reduction in female-derived macrophages. In general, reduction of M2-like macrophages emphasizes successful clearance of parasites, since survival of many infective agents is normally found in M2 macrophages [5,61,62].

The initial M1 polarization was also observed by Ontoria *et al*. when monitoring polarization in *L. infantum-*infected spleen cells of BALB/c mice [63], whereas in natural infections, VL normally correlates with high M2 polarization, characterized by increased arginase levels in the blood [64] and soluble CD163 in human [65,66] and canine VL [67]. This contradiction of polarization *in vitro* and *in vivo* could be partly explained by missing factors of the natural infection, such as sandfly saliva, which was shown to induce M2 polarization [68]. In line with this, and somewhat unexpectedly, we also did not observe any significant infection-dependent differences in Arginase-1 expression upon infection. This may reflect, or be a consequence of, the infection-associated reduction in M2-like macrophages.

Many cytokines can have dual effects during an infection with *Leishmania*, depending on the host as well as the infecting species and disease manifestation [69], and have been discussed as drivers for sex difference in parasitic diseases [70]. In this study, TNF, IL-10 and IL-8 showed an infection-dependent induction, with strong secretion at early time points but, surprisingly, without significant sex-specific differences. In contrast transcriptome analysis revealed relevant RNA-level differences that support sex differences in *Leishmania* infection. This distinct cytokine induction mirrors the important interaction of pro- and anti-inflammatory cytokines [8,32]. TNF is associated with host protection and parasite elimination, being a key cytokine for M1 polarization [71,72], and for the formation and maintenance of granuloma, in which parasites are encapsulated, thereby controlling the infection [73–76]. Elevated levels were shown in VL patients [75,77], as well as in *in vivo* rodent models of *L. donovani* infection [73]. Conversely to the results of this study, sex-specific secretion of TNF has been shown with a male predominance in murine *L. mexicana* infection [78], and Sellau *et al*. demonstrated TNF secretion to correlate with testosterone levels in human monocytes and mice [79]. IL-8 is another pro-inflammatory cytokine produced by leukocytes as well as many types of tissues upon inflammatory conditions, specifically activating and recruiting neutrophils [80]. Elevated levels of IL-8 have been observed in the serum of VL patients, which subsequently return to normal levels upon resolution of the disease, suggesting its potential as a circulating disease biomarker [81,82]. IL-10 promotes the anti-inflammatory, TH2-associated response that is beneficial for parasite survival and disease progression [83]. In animal models, increased IL-10 levels have been shown to be induced upon *Leishmania* infections, with higher levels reported in cutaneous lesions of male hamsters [21] and in murine male-derived macrophages *in vitro* [84]. In contrast, we observed a slight female bias in the expression of *IL10* at 6 hpi. The secretion of IL-18 was reduced after 6 hours of infection compared to uninfected cells in either sex, but no differences could be observed at later time points. IL-18 is a pro-inflammatory cytokine that contributes to host resistance against viscerotropic *Leishmania* species. Mice deficient in IL-18 exhibit significantly higher parasite burdens following infection with L. donovani, indicating a critical role for IL-18 in controlling visceral leishmaniasis [85]. Furthermore, IL-18 deficiency also results in increased susceptibility to infection with the dermotropic species *L. amazonensis*, suggesting that IL-18-mediated protection extends beyond viscerotropic parasites [86]. However, no epidemiological studies have reported on IL-18 levels and this cytokine has not been studied in the context of sex difference.

The strongest transcriptional response, based on the number of DEGs, was seen at early time points of infection, diminishing over time and correlating with the limitations of the *in vitro* infection assay and the clearance of the parasites. Nevertheless, the results indicated an early transcriptional regulation and strongly suggest the investigation of earlier time points in future *in vitro* studies or transcriptome profiling, complementing published work [87,88]. In the comparison of infected and uninfected macrophages of the same sex, KEGG pathway analysis revealed the induction of many immunity-related signaling pathways in both sexes at 6 and 16 hpi. These included pathways such as TNF signaling, NF-B signaling pathway, hypoxia- inducible factor 1 (HIF-1) signaling pathway and IL-17 signaling. These pathways were also found in other publications focusing on transcriptional profiling of infected macrophages [89–91]. Within these pathways, the most strongly regulated genes at 6 and 16 hpi coded for isoforms of the metallothionein family. The same tendencies were observed in the transcriptional profiling of macrophages infected with *L. major, L. amazonensis* and *L. chagasi* [88,91], and also in multiple other infections as reviewed by Dai *et al*. [92]. Genes encoding metallothionein family proteins are induced by a variety of metals or pro-inflammatory mediators, such as TNF or glucocorticoids and are involved in metal detoxification and protection against oxidative damage. Although the role of this protein family is not yet clear in the context of leishmaniasis, the expression is often associated with transmission of resistance through the regulation of cellular oxidative burst [93].

As with many other infections, such as in murine infection with *Plasmodium* spp. or bacterial infections [94,95], *HMOX1* was induced by the infection with *L. infantum* in both sexes, which is associated with pathogen survival within the macrophage. It has been shown that the expression of *HMOX1*, as shown for *L. donovani*, can be induced solely by surface molecules of the parasite [96]. The survival of pathogens is conferred by the protection from oxidative burst and reduced TNF levels and ROS production, as shown in HMOX1-/- mice, which present lower *L. chagasi* burdens compared with wild type mice [97]. Additionally, chemokine-encoding genes involved in the recruitment of immune cells such as *CXCL2*, *CXCL3* and *CCL20* were upregulated during the course of infection. These transcripts were also found in transcriptome analyses of spleen macrophages from *L. donovani*-infected hamsters [98] and can be correlated with either parasite killing or persistence through the attraction of other host cells [99,100].

Sex-specific expression was observed for several genes associated with type Ⅰ interferon signaling, indicated by higher expression of interferon stimulated genes (ISG) in female macrophages at 6 hpi, such as *OASL*, *MX1* and several genes encoding interferon-induced proteins (*IFIT2, IFIT3, IRF5*). Interferon signaling is known for its important role in the defense against viruses and their proliferation [101,102]. Interferon- signaling was also shown to be crucial for the defense against *Leishmania* infection and many other infectious diseases [103], and both type Ⅰ and Ⅱ levels have been described to act antagonistically to testosterone levels in naïve neutrophils and under infection [49,50]. However, type Ⅰ interferons have also gained interest in the context of protozoan parasite infections [104,105]. For *Leishmania* infections, the infecting strain, the timing and the concentration of the interferons thereby seemed to be important. In the visceral *in vivo* model using *L. donovani*, protection against the infection was conferred in a IRF7-dependent manner [106]. Similarly in cutaneous *L. major* infection, protection occurred through IFN--dependent induction of NOS2 [107]. Similar results were observed using an early treatment of murine peritoneal macrophages at a low dose *in vitro* [108]. In contrast to our findings and those of previous studies, *L. infantum* infection was associated with increased susceptibility mediated by type I interferon-induced IL-27 secretion, which inhibited a protective Th17 response [109]. Additionally, van Bockstal *et al.* reported that IFN-α stimulation led to increased Siglec-1 expression on bone marrow-derived macrophages, contributing to greater susceptibility to viscerotropic *Leishmania* species *in vitro* [110]. This is inconsistent with our findings, as we not only see an upregulation of type Ⅰ interferons in female macrophages associated with less severe infections with *L. infantum*, but also downregulation of *Siglec1* in both sexes. Differences in the expression of cytokine-encoding genes between *L. infantum* infected male and female macrophages were also observed. This was especially apparent for *IL15*, which was specifically expressed in female macrophages upon infection. This interleukin is associated with the suppression of IL-4 and induction of IL-12 secretion in human VL and therefore furthers the elimination of the parasite [111,112].

On the other hand*, CXCL1, IL32* and *IL-1β* were more highly expressed in male macrophages than in females at the initial time point. CXCL1 is induced after microbial infection and plays a key role in the recruitment of neutrophils and monocytes to the site of inflammation via CXCL2 signaling. The right balance is crucial, however, as insufficient egress may lead to failed microbial elimination, while excessive egress risks tissue damage [113]. In a prior investigation by our group, increased levels of CXCL1 were observed in classical monocytes from male mice, both under steady-state conditions and on day 3 of hepatic amebiasis, which led to an increased immunopathology caused by the recruitment of further immune cells. This male bias was similarly observed in human monocytes, with CXCL1 concentration positively correlating with testosterone levels [79]. IL-1 levels were also associated with disease progression due to immunopathology in patients with cutaneous leishmaniasis caused by *L. braziliensis* but did not affect phagocytosis or parasite killing [114]. Additionally, mice deficient for IL-1 or IL-1, respectively, were less susceptible to *L. major* infections, showed delayed and milder disease expression and increased TH1 polarization [115]. At 16 hpi, *NLRP1* and *NLRP3* were down-regulated in male-derived macrophages. As reviewed by Harrington and Gurung [116], NLRP3 inflammasome activation can have both protective and pathogenic effects in *Leishmania* infection. While it promotes IL-18 and IL-1β secretion, which may help control the infection, it can also lead to harmful outcomes—such as increased neutrophil recruitment and a TH2-biased immune response—that support parasite survival, particularly in *L. major* infection [117].

Further, genes associated with antibody response and B-cell activation were more strongly down-regulated in male macrophages, as seen by the GO term enrichment for biological processes and the top down-regulated genes. These processes comprised the genes *FCGR2A* and *FCGR2B*. The role of B-cells has not been studied in detail, but the presence and activation of polyclonal B-cells has been associated with disease exacerbation, as B-cell deficient mice showed later onset of symptoms, smaller lesions [118,119] and were able to avert splenomegaly [120]. On the other hand, it was shown in *L. major*-infected mice that IgG antibodies specifically increased antigen uptake by DCs via FCGR3 binding, leading to increased TH1 response by increased antigen presentation [121].

To analyze the effects of sex hormones on *L. infantum* infection, macrophages were treated with either E2 or DHT prior to infection. For estradiol treatment, no treatment-specific differences were observed for either sex both regarding infection rate and parasite burden at 28 hpi, although variations in the responsiveness to the treatment between female donors were observed. Similarly, supplementation of DHT did not alter the infection rate or parasite burden in either sex. However, in infection models with other *Leishmania* species, a clear influence of hormone treatment was observed. Treatment of female murine bone marrow-derived macrophages (BMMs) with estradiol subsequent to infection with *Leishmania mexicana* resulted in significantly reduced infection rates and a lower number of intracellular amastigotes per macrophage, highlighting the potential modulatory role of estradiol in enhancing the resistance of female macrophages, caused by enhanced IL-12p70 levels. On the other hand, treatment with DHT produced comparable protective effects in male BMMs, whereas treatment with estradiol in male BMMs not only failed to provide a protective effect but actually increased the infection rate [84]. Conversely, testosterone treatment of female BMMs and human peripheral blood mononucleated cells (PBMCs) following infection with *L. donovani* or *L. amazonensis*, respectively, led to increased infection rates in both and higher parasite loads in the case of *L. donovani* infection [28,122]. The influence of sex hormone treatment is highly variable and depends significantly on factors such as the infective agent, the donor’s sex, genetic background and the chosen treatment regimen applied, which varied extensively between the cited studies. These complexities may account for the differences observed across studies and highlight the importance of conducting further experimental investigations to clarify these interactions and their underlying mechanisms. Current limitations observed across our study as well as others include small donor cohort sizes and the use of heterogeneous steroid treatment protocols, which may confound results. Addressing these issues by employing larger, more diverse donor populations and standardized treatment regimens could significantly enhance the reliability and interpretability of future findings.

In summary, our findings present new insights into the biological determinants of the dimorphic outcome of leishmaniasis. The infection model based on human primary macrophages constitutes a powerful tool for the investigation of sex-based differences in the immune response following the infection with cutaneous and visceral *Leishmania* species. This method can be utilized for the investigation of sex-specific differences on the cellular level using high-content screening, the immunological and transcriptional level and subsequently for the validation of identified candidate molecules for treatment or treatment improvement of leishmaniasis. Additionally, this method can be expanded by incorporating additional immune cell types for a broader investigation of the immune response.

## Methods

### Human samples

Buffy coat samples from healthy donors aged 18–50 years of age were obtained from the Centre for Diagnostics, Institute for Transfusion Medicine, University Hospital Hamburg Eppendorf. Information on age (year of birth), sex, blood group and cytomegalovirus serological status was provided.

### *Leishmania* culture

All experiments were executed using *L. infantum* (strain MHOM/FR/91/LEM2259 clone 3511, expressing zymodeme MON-1) [123,124]. Parasites were routinely cultured in their promastigote stage at 25°C in supplemented M199 medium (Sigma-Aldrich, with Hank`s salts, 20% heat-inactivated fetal calf serum (FCS), 40 mM HEPES, 10 mg/L Haemin, 0.1 mM Adenine, 6 µM 6-Biopterin, 2 mM L-Glutamine, 100 U Penicillin and 100 µg/mL Streptomycin; pH 7.4). For *in vitro* infections, promastigotes were grown to early stationary phase. Promastigotes were counted using a Multisizer 3 Coulter counter (Beckmann Coulter).

### Isolation of human primary CD14^+^ monocytes

Following dilution of buffy coat samples at a ratio of 1:2 in PBS, isolation of human PBMCs from Buffy Coat samples was performed using 50 mL SepMate tubes (StemCell) according to manufacturer’s instructions. Residual erythrocytes in isolated cells were lysed using 1x RBC lysis buffer kit (BioLegend). The amount of PBMCs required for monocyte isolation were sedimented by centrifugation (5 min, 350 *g*, 4°C) and washed (5 min, 350 *g*, 4°C) with 2 mL MACS buffer (2 mM EDTA and 0,5% protease-free BSA in PBS). Supernatant was discarded and cells were resuspended in 50 μl vortexed -CD14 magnetic particles (BD Bioscience) per 1x10^7^ PBMCs and mixed thoroughly. After incubation for 30 min at room temperature (RT), cells were diluted to 1-8x10^7^ cells/mL in MACS buffer and subjected to magnetic pull-down for 10 min. The supernatant was carefully removed without disturbing the sediment. The cell pellet was resuspended twice in 1 mL MACS buffer and subjected to pull-down for 4 min. Following the second washing step, cold PBS was used to resuspend the cell pellet and to remove cells from magnet. After sedimentation (5 min, 350 *g*, 4°C), monocytes were resuspended in a suitable volume of pre-warmed, complemented RPMI 1640 medium (cRPMI; see below).

### Generation of human primary monocyte-derived macrophages

For the differentiation of human monocytes into hMDMs, CD14^+^-selected monocytes were cultured in multiwell plates according to the experimental design (1x10^5^ cells/well in 96- well Phenoplates ultra (revvity) for confocal high content screening; 5x10^5^ cells/well in 12-well plates for RNA isolation; 1x10^6^ cells/well in 6-well plates for flow cytometry staining) in cRPMI (PAN Biotech, with 10% heat-inactivated and charcoal-stripped FCS (to minimize the influence of external hormones [125]), 2 mM L-Glutamine, 100 U Penicillin and 100 µg/mL Streptomycin, 10 ng/mL M-CSF (BioLegend)) (96-well plate: 200 µL; 12-well plate: 2 mL; 6-well plate: 5 mL) at 37°C and 5% CO_2_. Half of the medium was carefully removed on day 4 and 6 after isolation and replaced with new pre-warmed medium, with the concentration of M-CSF always calculated for the full medium volume. Complete medium change was done at day 8 after isolation. Cells were used for *in vitro* infection on day 11 post isolation.

### *In vitro* polarization of macrophages

For the *in vitro* polarization of macrophages, CD14^+^ monocytes were differentiated in suitable well plates using cRPMI complemented using GM-CSF (M1 polarization) (10 ng/mL, BioLegend) or M-CSF (M2 polarization) for 7 days with standard media changes. M1 polarization was achieved by supplementing cRPMI + GM-CSF with 20 ng/mL IFN- (Sigma-Aldrich), whereas M2 polarization was induced by supplementing cRPMI + M-CSF with 20 ng/mL IL-4 (Sigma-Aldrich). Macrophages were polarized for 72 hours with supplemented media before immunofluorescent or flow cytometry staining [59,126,127].

### Steroid hormone treatment of macrophages

Experiments investigating the influence of steroid hormone treatment on *Leishmania* infection were conducted in media without phenol red to avoid external estrogen binding [125]. For the treatment of macrophages with steroid hormones, hMDMs were generated in 96-well Phenoplates using cRPMI (PAN Biotech). Mature macrophages (day 11 post isolation) from male or female donors were stimulated with 50 µL DHT (Merck) or E2 (Sigma-Aldrich) in the corresponding concentration (0.01-5 µM) for 30 min (37°C, 5% CO_2_). After incubation, *L. infantum* parasites were added directly (50 µL) without prior washing of the cells.

### Infection assay

Mature macrophages were infected with *L. infantum* stationary promastigotes at a multiplicity of infection (MOI) of 15:1 by pelleting the respective number of parasites (10 min, 1260 *g*, 4°C). Parasites were resuspended in a suitable volume of cRPMI (96-well plate: 100 µL/well; 12-well plate: 500 µL/well; 6-well plate: 1 mL/well) and added to the adherent macrophage layer. Macrophages were incubated with parasites for 4 h followed by three washing steps with warm PBS to remove the extracellular parasites. Macrophages were incubated at 37 °C and 5% CO_2_ for the required time period. To fix infected cells, supernatants were removed and cells were washed twice with warm PBS, before fixation with 4% paraformaldehyde (Thermo Fischer) for 25 min at RT. After fixation, microplates were stored at 4° C (200 μL PBS/well) until being further processed for immunofluorescence analysis.

### Labelling of PVs

Labelling of *Leishmania* spp.- induced PVs was done using LysoBrite Red DND-99, which specifically labels acidic compartments within cells, according to manufacturer’s instructions. Briefly, the medium of infected cells and respective controls was replaced by 100 µL cRPMI supplemented with LysoBrite Red DND-99 at 28 hpi. Cells were further incubated for 2 hrs before fixation and LysoBrite signal was detected using AF568 channel of confocal, high-content microscope Opera Phenix (revvity).

### Immunofluorescence staining

Immunofluorescent staining, image acquisition and analysis were performed as described previously [38]. Briefly, macrophages were stained with pan-*Leishmania* heat shock protein 90 (-*L*HSP90) antibody (1-1.5 h, 1:4000) [128,129] and -mouse-IgG coupled to Alexa Fluor (AF) 647 (Invitrogen by Thermo Fisher) (0.5-1 h, 1:8000) for detection of intracellular parasites. Cells were co-stained with DAPI (Sigma-Aldrich) for nuclear staining (0.5-1 h, 1:100).

### Image acquisition

Two-dimensional microscopy images were created using the confocal, high-content microscope Opera Phenix (revvity) with 20x water objective lens. Single cell detection was obtained in the DAPI channel (excitation: 405 nm; emission: 435–480 nm; exposure time: 350–400 ms; laser power: 100%); the parasites and host cell cytoplasm stained with -*L*HSP90-AF647 were detected in AF647 channel (excitation: 640 nm, emission, 650–760 nm; exposure time: 300 ms; laser power: 100%). Fluorescent images (15 fields per well) were acquired at seven different planes on the z-axis (first plane = -11 μm, last plane = 1 μm, distance between planes = 2 μm) to increase resolution. Using these settings, a cell confluency of approximately 80% per well was obtained, yielding 4000–6000 macrophages for quantification. In case of a confluency > 80%, but still a uniform monolayer of cells, the number and placement of images per well was adjusted (32 fields per well) and the obtained number of macrophages increased proportionally.

### Image and data analysis

Analysis of acquired microscopy pictures was done using image analysis sequence set up with the Harmony software (version 4.6) and the customized parameters described in Tab. S1. Briefly, the image analysis sequence is divided into different processing modules, called *building blocks* (S1 Table). In a first step, the input image was defined by merging all confocal images from fluorescent channels followed by the image segmentation to defined image objects. To calculate the viability of macrophages, the total number of cells was counted by the DAPI-stained cell nuclei using signal strength and size in the *find nuclei* building block. The cytoplasm of cells was defined by the detection of *L*HSP90-staining in the Alexa Flour fluorescent channel (*L*HSP90-AF647) using the *find cytoplasm* building block. For the detection of *Leishmania* parasites to calculate, for example, total parasite number or the proportion of infected macrophages, intracellular spots, defined by fluorescence signals in the DAPI and Alexa Flour channels within previously defined cell bodies, were detected using the *find spots* building block. The properties of detected objects were calculated (*Calculate Intensity Properties* and *Calculate Morphology Properties*) for all segments separately. The previously calculated properties were then used to differentiate between false-positive spots and parasites, as well as uninfected and infected cells within the *Select Population* building block. The final step was to use the *Define Results* building block to calculate and display the desired readout values and parameters, such as total number of macrophages and *Leishmania* parasites, % infected macrophages and mean number of parasites per infected macrophage. If applicable, quantification of the image analysis quality was assessed using Z´-value calculation as mentioned by Fehling *et al*. [38].

Some data sets were normalized to the respective control to calculate viability, infection rate and parasite burden. The normalized data were presented as percentage of control (POC) and calculated according to the following formula:


$$\text{Percentage of control}\left(\text{POC}\right)\text{=}\frac{Xd}{Xc}$$

POC = Percentage of data point in comparison to control.

Xd = Value of data point.

Xc = Respective mean value of control at data point.

### Cytokine analysis

For cytokine analysis, the supernatant of *L. infantum*-infected macrophages was analyzed using human customized flow-based LEGENDplex kit (BioLegend) according to manufacturer’s instructions (kit included: Arginase, CCL-17, CCL-2, CXCL-10, CXCL-8, IFN-, IL-10, IL-12p70, IL-18, IL-1, IL-23 and TNF-). Samples were measured using LSRⅡ or LSR Fortessa flow cytometer (BD Biosciences). Data analysis was performed

using LEGENDplex cloud-based analysis software (https://legendplex.qognit.com/user/login?next=home).

### Flow cytometry staining

The analysis of cell surface markers was performed as isolation control after PBMC isolation and magnetic selection of CD14^+^ monocyte. PBMCs and monocytes (1x10^6^ cells each) were stained with following antibodies, which were purchased at BioLegend, if not indicated otherwise: BV510-conjugated anti-CD14 (1:15, MSE2), FITC-conjugated anti-CD16 (1:15, 3G8) and BUV395-conjugated anti-HLA-DR (1:11, G46-6) (BD Biosciences).

For the staining of *L. infantum*-infected macrophages, at least 1x10^6^ cells were stained with the following antibodies (purchased at BioLegend, if not indicated otherwise): BV510-conjugated anti-CD14 (1:15, MSE2), FITC-conjugated anti-CD16 (1:15, 3G8), PerCP-Cy5.5-conjugated anti-CD163 (1:12.5, GHI/61), PECy7-conjugated anti-CD86 (1:15, IT2.2), PE-conjugated anti-CD200R (1:12.5, OX-108), APC Fire-conjugated anti-CD64 (1:12.5, 10.1) and APC-conjugated anti-CD206 (1:12.5, 15–2). Samples were measured using Cytek Aurora 5L spectral flow cytometer (Cytek Biosciences) and analyzed with FlowJo software (version 10).

### RNA sequencing and data analysis

3x10^6^ human primary macrophages per donor and condition were differentiated and infected with *Leishmania* parasites in 12-well plates. At required time points, cells were washed twice with pre-warmed PBS and collected in 500 μL TRIzol. Cell solutions were stored at -70°C until further processing. Samples were thawed on ice, and 500 μL of pre-warmed TRIzol was added, followed by centrifugation (10 min, 22,700 *g*, 4°C). The supernatant was transferred, mixed with 200 μL cold chloroform, and incubated for 3 min at room temperature. After centrifugation (30 min, 22,700 *g*, 4°C), the upper RNA-containing phase was collected, combined with 500 μL isopropanol, incubated for 10 min at room temperature, and centrifuged (15 min, 19,300 *g*, 4°C). The pellet was resuspended in 1 mL of undenatured 70% ethanol, centrifuged (5 min, 22,700 *g*, 4°C), dried at 56°C, and resuspended in 100 µL RNAse-free water. Further purification of the samples was performed using the NucleoSpin RNA clean-up Kit (Machery-Nagel) according to the manufacturer’s instructions. Samples were eluted in 100 µL RNAse-free water and RNA quality and concentration were measured by on-chip automated electrophoresis using Agilent 2100 Bioanalyzer with Agilent RNA 6000 Pico Kit and 2100 Expert Software (Agilent Technologies). Library preparation was performed using QIAseq Stranded mRNA Lib Kit UDI-A (Qiagen) and libraries were sequenced using NextSeq 1000/2000 P2 Reagents (200 Cycles) Kit v3 on the Illumina NextSeq1000/2000 system (Illumina) generating 100 bp paired-end reads or using a NextSeq500/550 Mid Output Kit v2.5 (150 Cycles) on the Illumina NextSeq500/550 system (Illumina) generating 75 bp paired-end reads.

RNA-sequencing raw data were processed using the Nextflow RNAseq (v.3.8) pipeline. Quality control of raw data was done with FastQC and quality reports were generated using MultiQC (v1.11). Trimmed reads were aligned to human genome GRCh38 obtained from iGenomes (s3://ngi-igenomes/igenomes/Homo_sapiens/NCBI/GRCh38) as provided by the Nextflow RNAseq pipeline. Expression data were normalized using variance stabilizing transformation (VST) in R package DESeq2 (v1.34). Relation between samples of different conditions (sex, infection state and time points) were evaluated using principal component analysis (PCA). This was computed on VST data using the scikit-learn (v1.2) Python package. Differential expression analyses between different conditions within time points or across time were conducted with DESeq2 (v1.34) package and multiple testing correction was done using Benjamini-Hochberg method. A comparison was defined as significant with an adjusted p-value < 0.05. The sequencing batch was used as a covariant in the statistical model to account for batch effects. Further visualizations were performed on VST normalized data. Volcano plots were created with R package EnhancedVolcano (v1.12). Heatmaps were created with freely available tool Heatmapper (http://heatmapper.ca/). Venn diagrams were created using online tool InteractiVenn (http://www.interactivenn.net/). Kyoto Encyclopedia of Genes and Genomes (KEGG) pathways analysis and GO-term enrichment analysis of all significantly different expressed genes or genes that passed the log_2_fold change threshold indicated in the figure legend were conducted using shinyGO (v0.8) (http://bioinformatics.sdstate.edu/go/) with default settings. All genes that were detected in the transcriptome were set as background. Database used for analyses are indicated in the figure legends.

### Statistical analysis

Statistical analyses were carried out using GraphPad Prism software (v. 10.0.1). All data were tested for Gaussian distribution using the Shapiro-Wilk test and further analyzed using parametric or non-parametric versions of statistical tests and appropriate *p*-value adjustments indicated in the respective figure legends. Significances are shown in graphics as follows: *p < 0.05; **p < 0.01; ***p < 0.001; ****p < 0.0001).

## Supporting information

S1 Fig Sex difference in the infection of human macrophages with *L. infantum.* Mature macrophages from female and male human blood donors were infected with *L. infantum* metacyclic promastigotes (MOI of 15:1) and infection parameters were quantified using Opera Phenix confocal microscope and customized image analysis sequence (S1 Table). Depicted are (A) % infected macrophages and (B) *Leishmania* parasites per infected macrophage with respective uninfected control subtracted within each sex. n_F/M 4hpi_ = 6/6, n_F/M 6hpi_ = 4/4, n_F/M 16hpi_ = 10/8, n_F/M 28hpi_ = 19/17, n_F/M 52hpi_ = 11/11, n_F/M 76hpi_ = 10/10, n_F/M 100hpi_ = 6/6. P-values were calculated using ordinary One-way ANOVA and Dunnett´s multiple comparison correction for time course within one sex and unpaired and two-tailed unpaired Student ´s t-test for comparison between sexes (*p < 0.05, **p < 0.01, ***p < 0.001, ****p < 0.0001).
(TIF)

S2 Fig Morphology-based distinction and phenotypic characterization of M1- and M2 polarized macrophages. Monocytes were differentiated into macrophages using media complemented with either M-CSF or GM-CSF. On day 7 post isolation, cells were polarized into M1-like macrophages using GM-CSF and IFN- or M2-like macrophages using M-CSF and IL-4. M0 macrophages were continuously cultivated using M-CSF. Mature Macrophages were fixed at day 11. (A) Representative images of differently polarized macrophages. (a,e,i) DIC pictures were acquired with EVOS FL Auto Fluorescence microscope. (b,f,j) Immunofluorescent pictures acquired with Opera Phenix confocal microscope. DAPI nuclear staining (blue) and cytoplasm with -*L*HSP90 coupled to AF647 (red). Images were analyzed with customized image analysis sequence (Harmony software) and the outcome for detection of (c,g,k) M1-like macrophages and (d,h,l) M2-like macrophages (green = positive, red = negative) is depicted. For panels (a-d) M1 macrophages, (e-h) M2-like macrophages and (i-l) M0-like macrophages were used. Scale bar = 100 μM and scale bar = 20 μM in close-up images. (B) Parameters established within image analysis sequence for morphological distinction between M1-like and M2-like macrophages. (C) Quantification of polarized macrophages detected within different analysis sequences. Data shown as boxplot, n = 5. P-values were calculated using two-tailed unpaired Student ´s t-test. (D) MFI of M1- or M2- related surface markers on CD14^+^ M1-like or M2-like polarized macrophages (using gating scheme Fig 2D). Data shown as boxplot, n = 4. P-values were calculated using two-tailed paired Student ´s t-test. (*p < 0.05, **p < 0.01, ***p < 0.001, ****p < 0.0001). DIC = differential interference contrast. Created in BioRender. Lotter, H. (2025) https://BioRender.com/jb9x5an.
(TIF)

S3 Fig Cytokine profile of *L. infantum* infected macrophages derived from men and women. Mature macrophages from female and male donors were infected with *L. infantum (*MOI 15:1) for 6–52 hours. Cytokines were determined in the culture supernatant using cytometric bead assay (LEGENDplex). Time-course analysis of the MFI of (A) IL-1 (B) CXCL10 (C) IL-23 (D) Arginase (E) CCL17 and (F) IL-12p70 at indicated time points (left side) and respective area under the curve (AUC) (right side). Data represent mean ± SEM, n_F/M _= 4/4.
(TIF)

S4 Fig Principal component analysis of *L. infantum*-infected macrophages from female and male donors. Mature macrophages from women and men were infected with *L. infantum* (MOI 15:1) for indicated time periods. mRNA from infected macrophages and naïve controls was isolated and subjected to whole transcriptome sequencing. PCA of sequenced uninfected and infected macrophages according to infection status (node color) and donor (node shape) at different time points after infection from (A) female-derived and (B) male-derived macrophages. Variance is depicted as percentage for both components.
(TIF)

S5 Fig Transcriptional analysis of *L. infantum*-infected macrophages from female and male donors 16 hpi. Mature macrophages from (A-D) women and (E-H) men were infected with *L. infantum* (MOI 15:1) for 16 hours. mRNA from infected macrophages and naïve controls was isolated and subjected to whole transcriptome sequencing. (A,E) Volcano plots depicting differential gene expression between uninfected and infected macrophages according to magnitude of change (Log_2_(fold change)) and statistical significance of change (-Log_10_(adjusted p-value). Genes significantly regulated between the conditions (Log_2_(fold change) > I0.25I, adj. p < 0.05) are marked in red (left side = low in infection, right side = high in infection). (B, F) Heatmaps depict top 30 regulated genes. (D, H) KEGG pathway analysis of significantly up-regulated genes in infection (adj p < 0.05, log2 (fold change) >1) and (C, G) Biological processes GO term enrichment of significantly down-regulated genes in infection (adj. p < 0.05, log2 (fold change) <-1). (C, D, G, H) Shown are the top 20 pathways/processes sorted by fold enrichment. Significances of enrichment indicated by color and number of genes in pathway indicated in node size.
(TIF)

S6 Fig Transcriptional analysis of *L. infantum*-infected macrophages from female and male donors. Mature macrophages from (A,B,E,F) women and (C,D,G,H) men were infected with *L. infantum* (MOI 15:1) for indicated time period. mRNA from infected macrophages and naïve controls was isolated and subjected to whole transcriptome sequencing. (B,D,F,H) Biological processes GO term enrichment of significantly up-regulated genes in infection (adj p < 0.05, log2 (fold change) >1) and (A,C,E,G) KEGG pathway analysis of significantly down-regulated genes in infection (adj. p < 0.05, log2 (fold change) <-1). Shown are the top 20 pathways/processes sorted by fold enrichment. Significances of enrichment indicated by color and number of genes in pathway indicated in node size.
(TIF)

S7 Fig Sex differences in transcriptional analysis of *L. infantum*-infected macrophages. Mature macrophages from female and male donors were infected with *L. infantum* (MOI 15:1) for 16 hours. mRNA from infected macrophages and naïve controls was isolated and subjected to whole transcriptome sequencing. (A-F) Top enriched pathways identified using Reactome database depicted at the side of the heatmap. Heatmaps depict selected corresponding genes for (A) exclusively up-regulated genes in female samples, (B) similarly up-regulated genes in both female and male samples and (C) exclusively up-regulated genes in male samples, (D) exclusively down-regulated genes in female samples, (E) similarly down-regulated genes in both female and male samples and (F) exclusively down-regulated genes in male samples.
(TIF)

S8 Fig Influence of 5-Dihydrotestosterone (DHT) stimulation on L. infantum infection of macrophages. Mature macrophages from female and male donors were stimulated with 5-Dihydrotestosterone (DHT) in various concentrations (0.01– 5μM) before infection with *L. infantum* (MOI 15:1) for 28 hours. Parameters were quantified using Opera Phenix confocal microscope and customized image analysis sequence. (A) Infection rate and (B) parasite burden of macrophages normalized to corresponding infected unstimulated control (POC = 100). Data are shown as boxplot, DHT = n_F/M -_ = 3/3.
(TIF)

S1 Table Parameters for customized automated image analysis sequence established in Harmony software (v. 4.6). (PDF)

S2 Table Differentially expressed gene list for female *L. infantum*-infected human macrophages at different time points of infection. (XLSB)

S3 Table Differentially expressed gene list for male *L. infantum*-infected human macrophages at different time points of infection. (XLSB)
