# Supplementary figures and images for: Transcriptome profiling of L. infantum-infected human macrophages reveals sex-specific type I interferon induction
Source: PLoS Pathog. 2025 Aug 12;21(8):e1013427. doi: 10.1371/journal.ppat.1013427 (PMC12367141; doi:10.1371/journal.ppat.1013427)

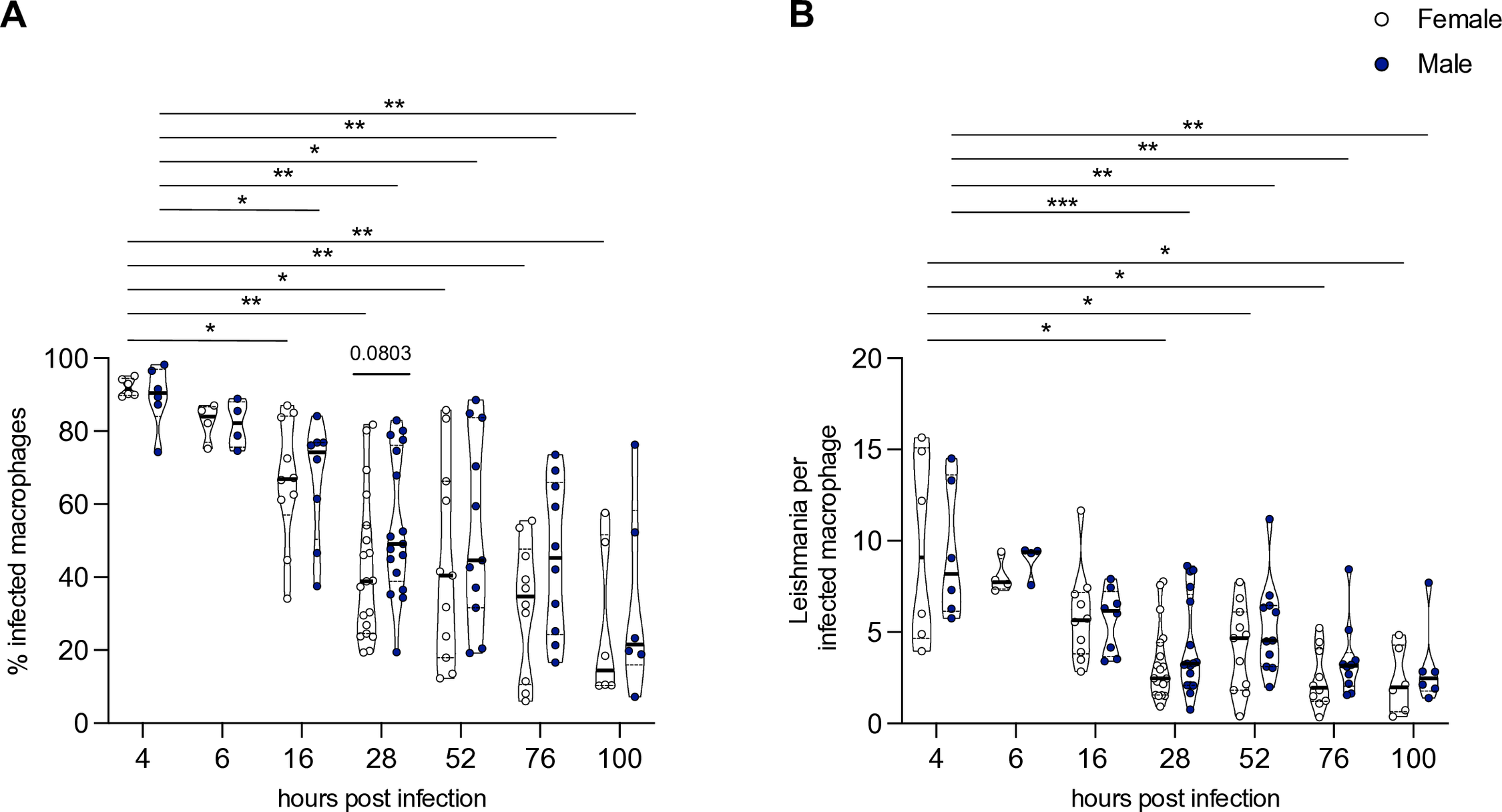

Supplement: S1 Fig — Mature macrophages from female and male human blood donors were infected with L. infantum metacyclic promastigotes (MOI of 15:1) and infection parameters were quantified using Opera Phenix confocal microscope and customized image analysis sequence (S1 Table). Depicted are (A) % infected macrophages and (B) Leishmania parasites per infected macrophage with respective uninfected control subtracted within each sex. nF/M 4hpi = 6/6, nF/M 6hpi = 4/4, nF/M 16hpi = 10/8, nF/M 28hpi = 19/17, nF/M 52hpi = 11/11, nF/M 76hpi = 10/10, nF/M 100hpi = 6/6. P-values were calculated using ordinary One-way ANOVA and Dunnett´s multiple comparison correction for time course within one sex and unpaired and two-tailed unpaired Student ´s t-test for comparison between sexes (*p < 0.05, **p < 0.01, ***p < 0.001, ****p < 0.0001). (TIF) [file ppat.1013427.s001.tif]

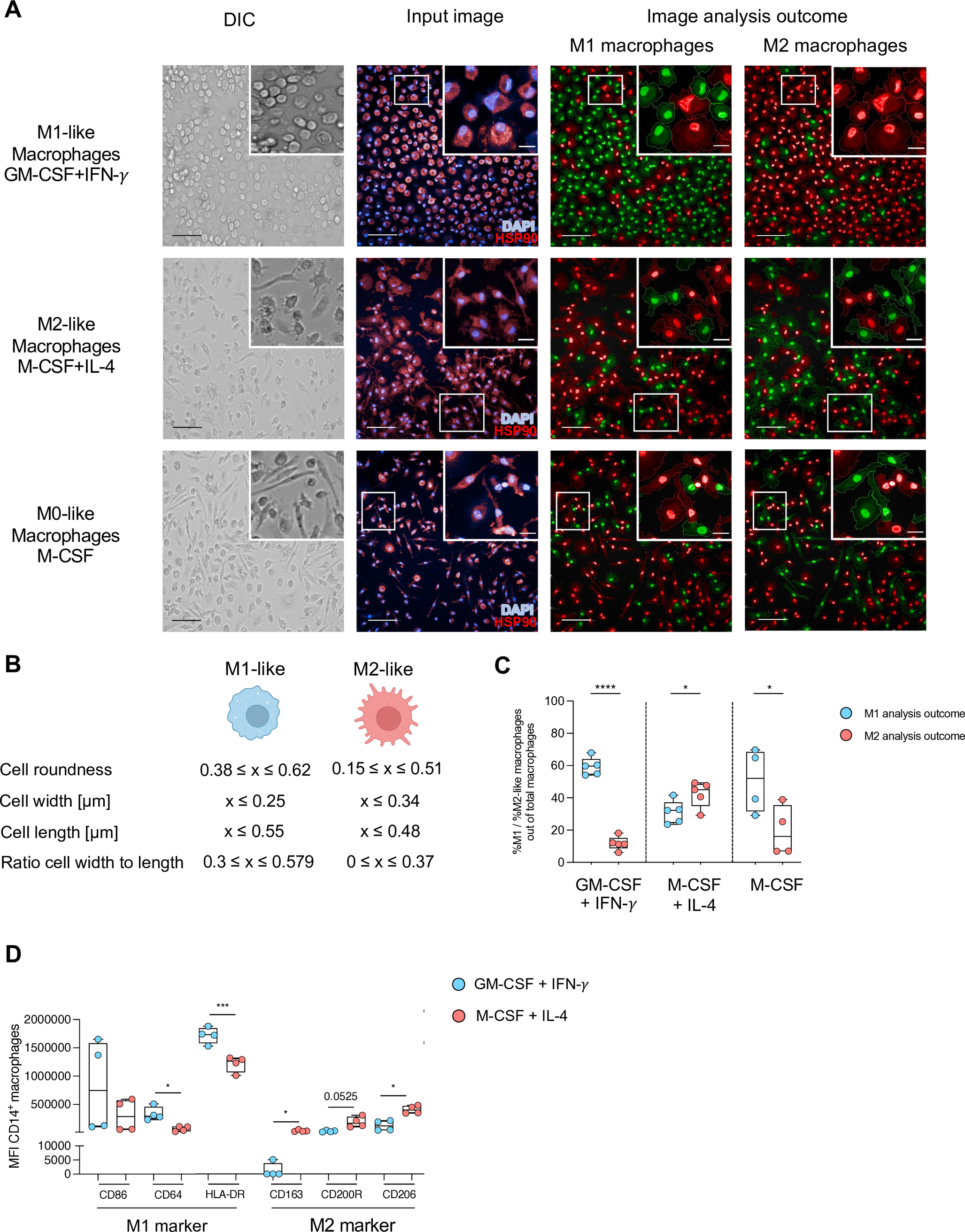

Supplement: S2 Fig — Morphology-based distinction and phenotypic characterization of M1- and M2 polarized macrophages. Monocytes were differentiated into macrophages using media complemented with either M-CSF or GM-CSF. On day 7 post isolation, cells were polarized into M1-like macrophages using GM-CSF and IFN- or M2-like macrophages using M-CSF and IL-4. M0 macrophages were continuously cultivated using M-CSF. Mature Macrophages were fixed at day 11. (A) Representative images of differently polarized macrophages. (a,e,i) DIC pictures were acquired with EVOS FL Auto Fluorescence microscope. (b,f,j) Immunofluorescent pictures acquired with Opera Phenix confocal microscope. DAPI nuclear staining (blue) and cytoplasm with -LHSP90 coupled to AF647 (red). Images were analyzed with customized image analysis sequence (Harmony software) and the outcome for detection of (c,g,k) M1-like macrophages and (d,h,l) M2-like macrophages (green = positive, red = negative) is depicted. For panels (a-d) M1 macrophages, (e-h) M2-like macrophages and (i-l) M0-like macrophages were used. Scale bar = 100 μM and scale bar = 20 μM in close-up images. (B) Parameters established within image analysis sequence for morphological distinction between M1-like and M2-like macrophages. (C) Quantification of polarized macrophages detected within different analysis sequences. Data shown as boxplot, n = 5. P-values were calculated using two-tailed unpaired Student ´s t-test. (D) MFI of M1- or M2- related surface markers on CD14+ M1-like or M2-like polarized macrophages (using gating scheme Fig 2D). Data shown as boxplot, n = 4. P-values were calculated using two-tailed paired Student ´s t-test. (*p < 0.05, **p < 0.01, ***p < 0.001, ****p < 0.0001). DIC = differential interference contrast. Created in BioRender. Lotter, H. (2025) https://BioRender.com/jb9x5an. (TIF) [file ppat.1013427.s002.tif]

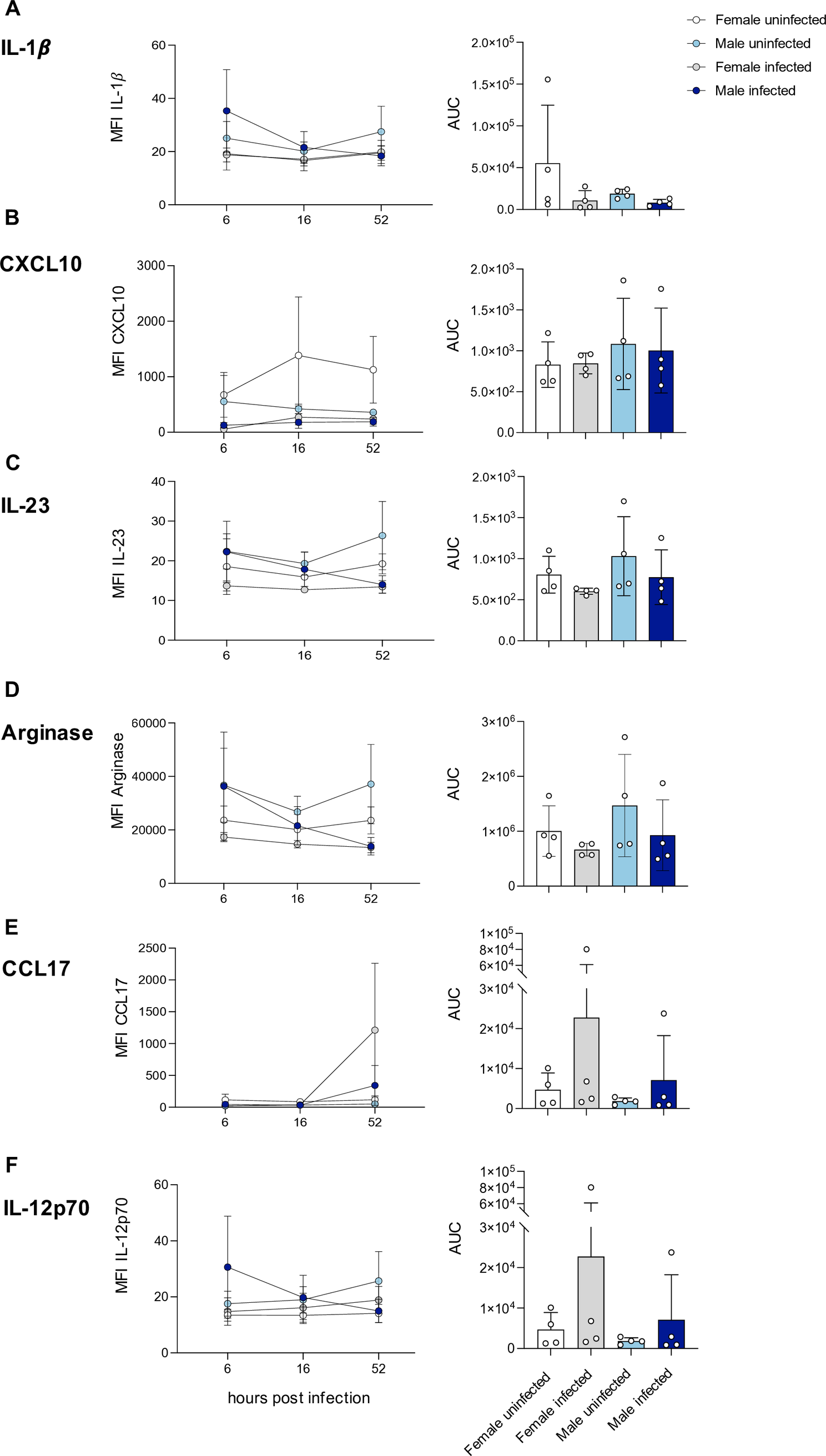

Supplement: S3 Fig — Mature macrophages from female and male donors were infected with L. infantum (MOI 15:1) for 6–52 hours. Cytokines were determined in the culture supernatant using cytometric bead assay (LEGENDplex). Time-course analysis of the MFI of (A) IL-1 (B) CXCL10 (C) IL-23 (D) Arginase (E) CCL17 and (F) IL-12p70 at indicated time points (left side) and respective area under the curve (AUC) (right side). Data represent mean ± SEM, nF/M = 4/4. (TIF) [file ppat.1013427.s003.tif]

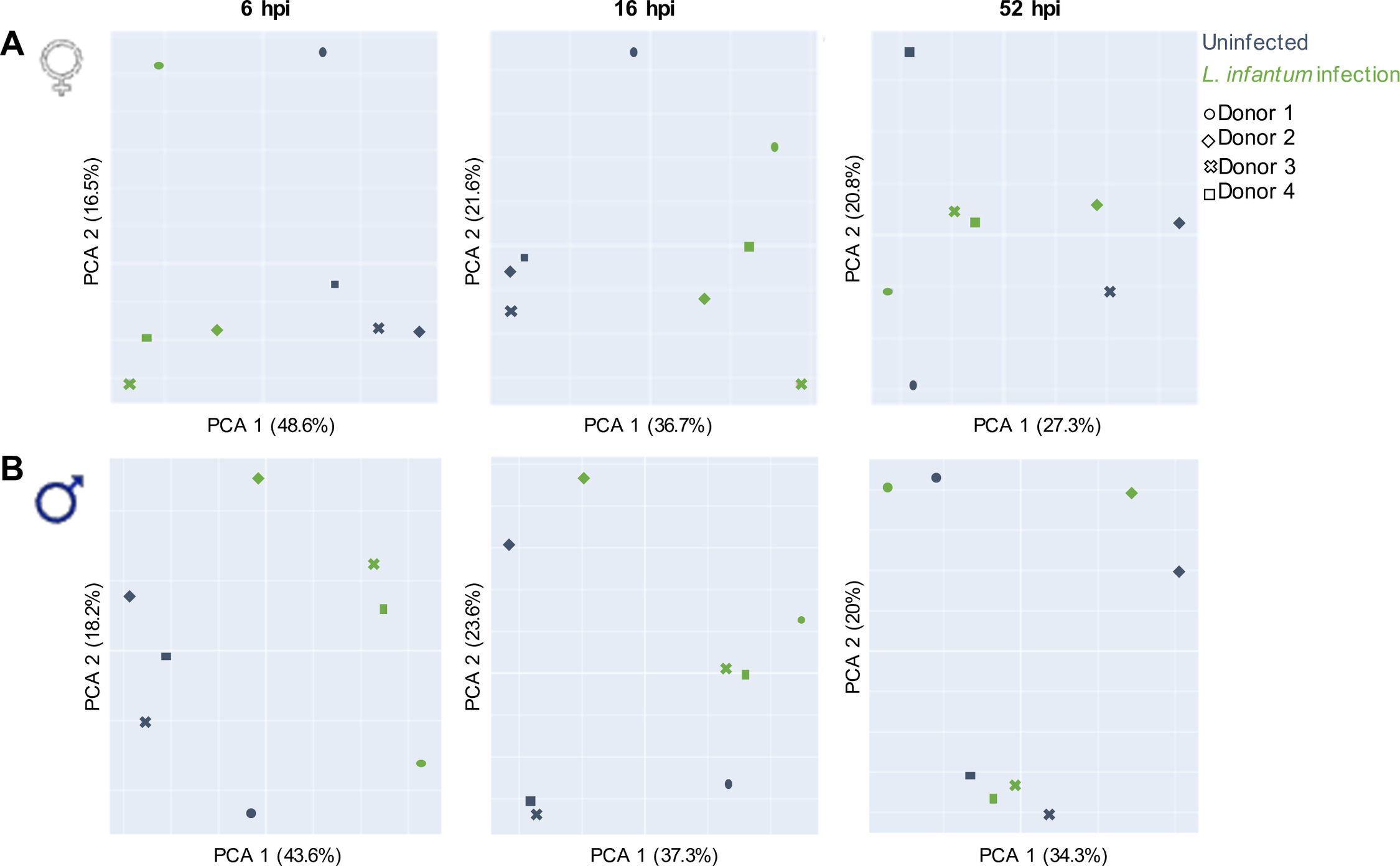

Supplement: S4 Fig — Mature macrophages from women and men were infected with L. infantum (MOI 15:1) for indicated time periods. mRNA from infected macrophages and naïve controls was isolated and subjected to whole transcriptome sequencing. PCA of sequenced uninfected and infected macrophages according to infection status (node color) and donor (node shape) at different time points after infection from (A) female-derived and (B) male-derived macrophages. Variance is depicted as percentage for both components. (TIF) [file ppat.1013427.s004.tif]

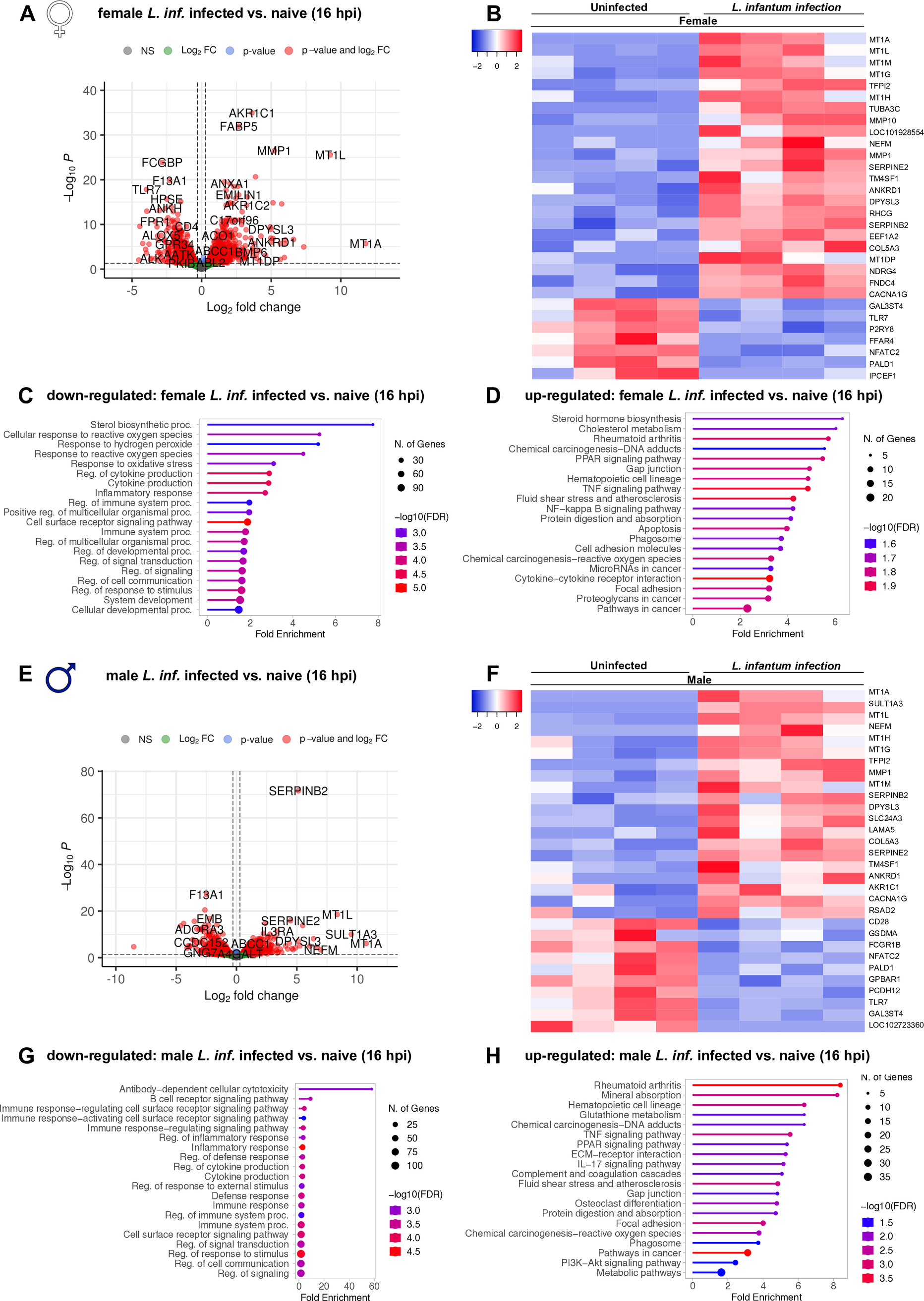

Supplement: S5 Fig — Mature macrophages from (A-D) women and (E-H) men were infected with L. infantum (MOI 15:1) for 16 hours. mRNA from infected macrophages and naïve controls was isolated and subjected to whole transcriptome sequencing. (A,E) Volcano plots depicting differential gene expression between uninfected and infected macrophages according to magnitude of change (Log2(fold change)) and statistical significance of change (-Log10(adjusted p-value). Genes significantly regulated between the conditions (Log2(fold change) > I0.25I, adj. p < 0.05) are marked in red (left side = low in infection, right side = high in infection). (B, F) Heatmaps depict top 30 regulated genes. (D, H) KEGG pathway analysis of significantly up-regulated genes in infection (adj p < 0.05, log2 (fold change) >1) and (C, G) Biological processes GO term enrichment of significantly down-regulated genes in infection (adj. p < 0.05, log2 (fold change) <-1). (C, D, G, H) Shown are the top 20 pathways/processes sorted by fold enrichment. Significances of enrichment indicated by color and number of genes in pathway indicated in node size. (TIF) [file ppat.1013427.s005.tif]

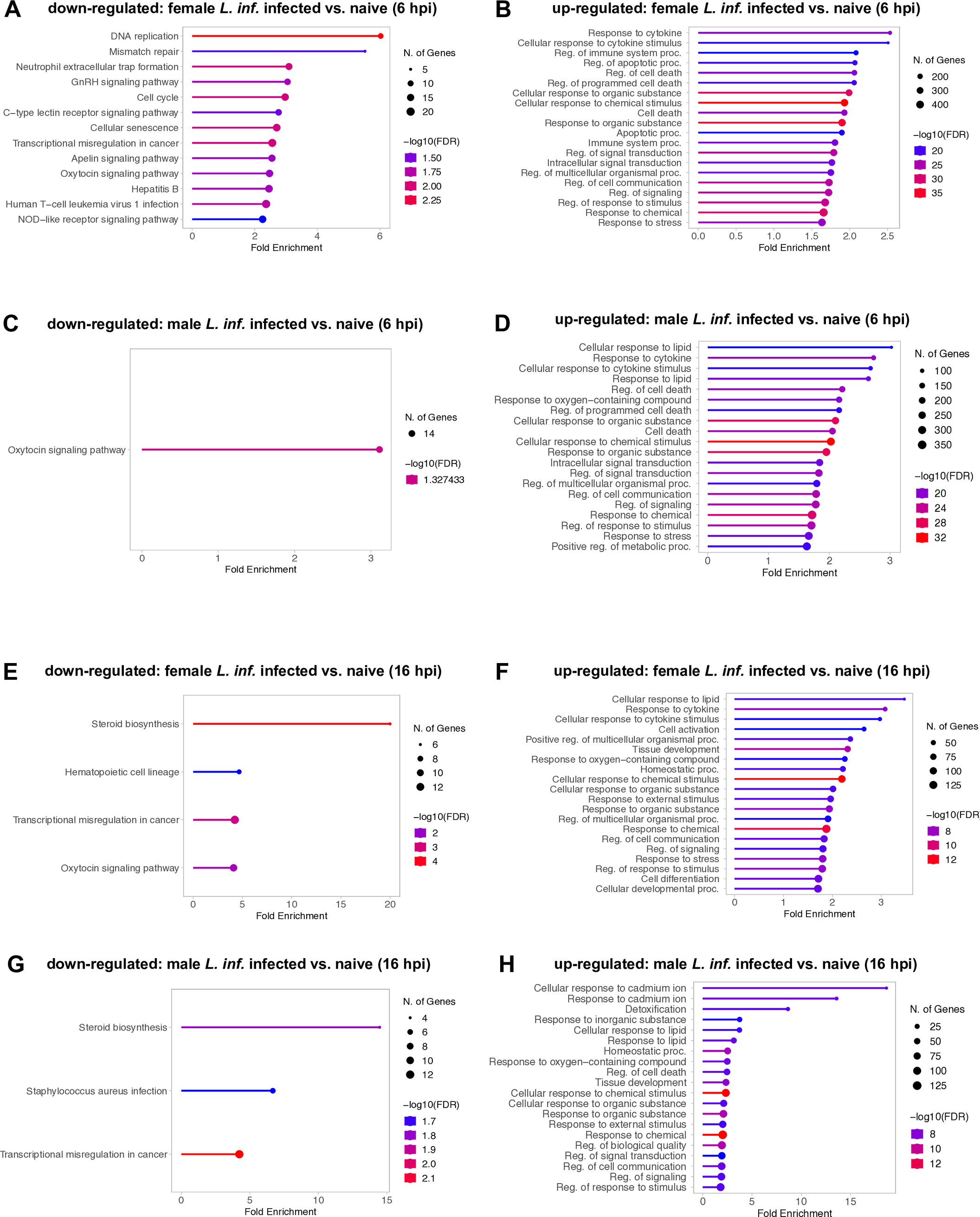

Supplement: S6 Fig — Mature macrophages from (A,B,E,F) women and (C,D,G,H) men were infected with L. infantum (MOI 15:1) for indicated time period. mRNA from infected macrophages and naïve controls was isolated and subjected to whole transcriptome sequencing. (B,D,F,H) Biological processes GO term enrichment of significantly up-regulated genes in infection (adj p < 0.05, log2 (fold change) >1) and (A,C,E,G) KEGG pathway analysis of significantly down-regulated genes in infection (adj. p < 0.05, log2 (fold change) <-1). Shown are the top 20 pathways/processes sorted by fold enrichment. Significances of enrichment indicated by color and number of genes in pathway indicated in node size. (TIF) [file ppat.1013427.s006.tif]

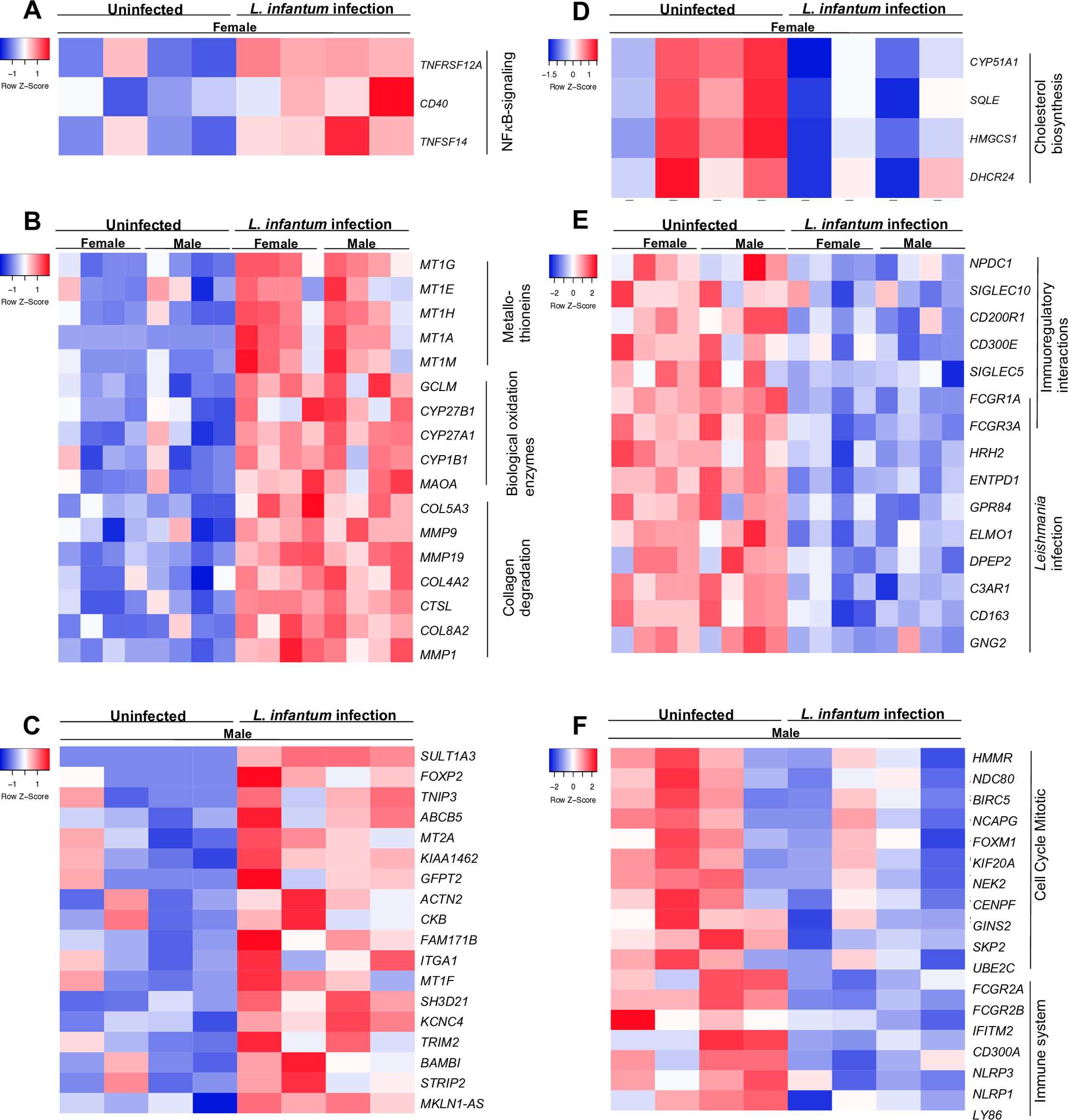

Supplement: S7 Fig — Mature macrophages from female and male donors were infected with L. infantum (MOI 15:1) for 16 hours. mRNA from infected macrophages and naïve controls was isolated and subjected to whole transcriptome sequencing. (A-F) Top enriched pathways identified using Reactome database depicted at the side of the heatmap. Heatmaps depict selected corresponding genes for (A) exclusively up-regulated genes in female samples, (B) similarly up-regulated genes in both female and male samples and (C) exclusively up-regulated genes in male samples, (D) exclusively down-regulated genes in female samples, (E) similarly down-regulated genes in both female and male samples and (F) exclusively down-regulated genes in male samples. (TIF) [file ppat.1013427.s007.tif]

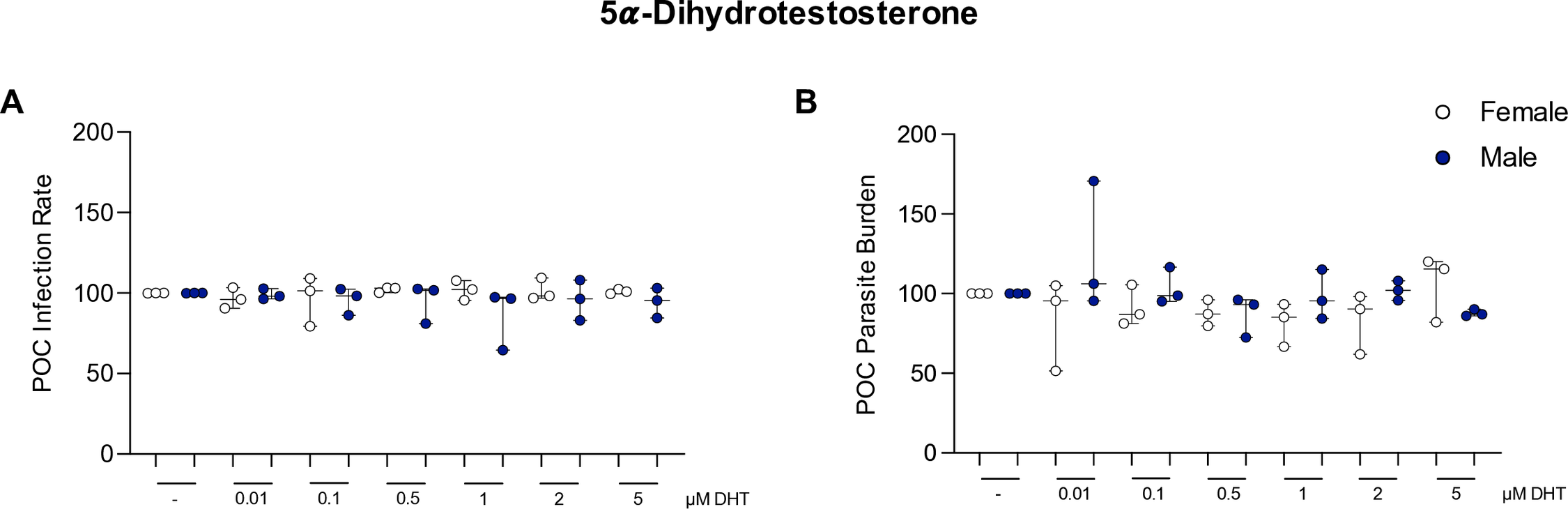

Supplement: S8 Fig — Mature macrophages from female and male donors were stimulated with 5-Dihydrotestosterone (DHT) in various concentrations (0.01– 5μM) before infection with L. infantum (MOI 15:1) for 28 hours. Parameters were quantified using Opera Phenix confocal microscope and customized image analysis sequence. (A) Infection rate and (B) parasite burden of macrophages normalized to corresponding infected unstimulated control (POC = 100). Data are shown as boxplot, DHT = nF/M - = 3/3. (TIF) [file ppat.1013427.s008.tif]
