# Supplementary material for: Transcriptome profiling of L. infantum-infected human macrophages reveals sex-specific type I interferon induction
Source: PLoS Pathog. 2025 Aug 12;21(8):e1013427. doi: 10.1371/journal.ppat.1013427 (PMC12367141; doi:10.1371/journal.ppat.1013427)
Supplement: S1 Table — (PDF) [file ppat.1013427.s009.pdf]

Analysis building block	Parameters	Objective
**Define Input**		
Input Image	Channel: DAPI, AF647<br>Flatfield correction: None<br>Stack processing: Maximum projection	Merging of images from different fluorescence channels and stacks
**Find Objectives**		
Find Nuclei	Channel: DAPI<br>ROI: None<br>Method: B<br>Common threshold: 0.02<br>Area: > 40 μm^2<br>Splitting coefficient: 19.9<br>Individual threshold: 0.14<br>Contrast: > -0.71<br><br>**Output population:** Macrophages	Image segmentation:<br><br>Determination of nuclei in host cells
Find Cytoplasm	Channel: AF647<br>Nuclei: Macrophages<br>Method: A<br>Individual threshold: 0.06<br><br>**Output region:** Cell, Cytoplasm, Membrane	Image segmentation:<br><br>Defining single cells by determination of host cell cytoplasm
**Calculate Object properties**		
Calculate Intensity properties	Channel: DAPI<br>Population: Macrophages<br>Region: Nucleus<br>Method: Standard<br>Calculate: Mean<br>**Property Prefix:** Intensity Nucleus DAPI<br><br>**Output Property:** Intensity Nucleus DAPI Mean	Quantification of properties inside defined regions and removal of borders
	Channel: AF647<br>Population: Macrophages<br>Region: Cytoplasm<br>Method: Standard<br>Calculate: Mean<br>**Property Prefix:** Intensity Cytoplasm AF647<br><br>**Output Property:** Intensity Cytoplasm AF647 Mean	
Calculate Morphology properties	Channel: AF647<br>Population: Macrophages<br>Region: Cytoplasm<br>Method: Standard<br>Calculate: Area [µm], Roundness, Width [µm], Length [µm], Ratio Width to Length<br>**Property Prefix:** M1 Cytoplasm<br><br>**Output Property:** Standard	
	Channel: AF647<br>Population: Macrophages<br>Region: Cytoplasm<br>Method: Standard (deprecated)	

	Calculate: Area [µm], Roundness, Width [µm], Length [µm], Ratio Width to Length<br>**Property Prefix:** M2 Cytoplasm<br><br>**Output Property:** Standard	
	**Find intracellular spots**	
Find Spots	Channel: AF647<br>ROI: Macrophages<br>Cell<br>Method: B<br>Detection Sensitivity: 0.11<br>Splitting Sensitivity: 0.844<br>Individual threshold: 0.06<br><br>**Output population:** Spots	
Calculate intensity properties	Channel: DAPI<br>Population: Spots<br>Region: Spot<br>Method: Standard<br>Calculate: Mean<br>**Property Prefix:** Intensity Spot DAPI<br><br>**Output Property:** Intensity Spot DAPI Mean	
	Channel: AF647<br>Population: Spots<br>Region: Spot<br>Method: Standard<br>Calculate: Mean<br>**Property Prefix:** Intensity Spot AF647<br><br>**Output Property:** Intensity Spot AF647 Mean	
Calculate Morphology properties	Channel: AF647<br>Population: Spots<br>Region: Spot<br>Method: Standard<br>Calculate: Area [µm], Roundness<br>**Property Prefix:** Spot<br><br>**Output Property:** Standard	
Calculate properties	Population: Spots<br>Method: By Formula<br>Formula: A/B<br>Variable A: Intensity Spot AF647 Mean<br>Variable B: Intensity Spot DAPI Mean<br><br>**Output Property:** AF/DAPI Ratio	
	**Select population of objects**	
Select population I (interesting spots)	Population: Spots<br>Method: Filter by Properties<br>Parameter: Spot Roundness ≤ 1.2<br>Spot Roundness > 0.25<br>Spot Area [px^2] 15 ≤ X ≤ 900<br>Relative Spot Intensity > 0.4<br>Spot Contrast > 0.1<br>Region Intensity >150<br>Corrected Spot Intensity: **must be adapted, staining-dependent**<br><br>**Output Population:** Interesting spots	Identification of population:<br><br>Discrimination of interesting, possibly parasite-based spots, from total spots

Select population Ⅱ (*Leishmania* parasites)	Population: Interesting Spots<br>Method: Linear classifier<br>Number of classes= 2<br>Parameter: Spot Roundness<br>Spot Area [px^2]<br>Spot Contrast<br>Relative Spot Intensity<br>Region Intensity<br>Spot to Region Intensity<br>Spot Background Intensity<br>Corrected Spot Intensity<br>Uncorrected Spot Intensity<br>Intensity Spot AF647 Mean<br>Intensity Spot DAPI Mean<br><br>**Output Population A:** Leishmania selected<br>**Output Population B:** false-positive	Identification of population:<br><br>Discrimination of parasites from unspecific intracellular spots
	Population: interesting spots<br>Method: Filter by property<br>Parameter: Regression A-B < -3	
	Population: Macrophages<br>Method: By related population<br>Related population: *Leishmania*<br>Parameter: Number of *Leishmania*<br>Mean Spot Area [px^2]<br>Mean Spot Roundness<br>Mean Spot Contrast<br>Mean Relative Spot Intensity<br>Mean Region Intensity<br>Mean Spot to Region Intensity<br>Mean Spot Background Intensity<br>Mean Corrected Spot Intensity<br>Mean Uncorrected Spot Intensity<br>Mean Intensity Spot AF647 Mean<br>Mean Intensity Spot DAPI Mean<br>Mean AF/DAPI Ratio<br>Mean Regression A-B<br>Mean Leishmania selected<br>Mean false-positive<br>Property Suffix: per Cell<br><br>**Output Properties:** By related Population	
Select population Ⅲ (infected macrophages)	Population: Macrophages<br>Method: Filter by Property<br>Parameter: Number of Leishmania per cell >0<br><br>**Output Population:** infected macrophage	Identification of population:<br><br>Discrimination of infected from uninfected macrophages
	Population: Macrophages<br>Method: Filter by Property<br>Parameter: Number of Leishmania per cell >2<br><br>**Output Population:** double infected macrophage	
	Population: Macrophages<br>Method: Filter by Property<br>Parameter: Number of Leishmania per cell >3<br><br>**Output Population:** massive infected macrophage	
	Population: Macrophages<br>Method: Filter by Property	Identification of population:

Select population Ⅳ (macrophage polarisation)	Parameter: M1 Cytoplasm Roundness 0.38≤X≤0.62<br>M1 Cytoplasm Length [µm] ≤ 55<br>M1 Cytoplasm Width [µm] ≤ 25<br>M1 Cytoplasm Ratio Length to Width 0.3≤X≤0.579<br><br>**Output Population:** M1 macrophage	Discrimination of polarisation state of macrophages based on their morphology
	Population: M1 Macrophages<br>Method: Filter by Property<br>Parameter: Number of Leishmania per cell >0<br><br>**Output Population:** infected M1 macrophage	
	Population: Macrophages<br>Method: Filter by Property<br>Parameter: M2 Cytoplasm Roundness 0.15≤X≤0.51<br>M2 Cytoplasm Length [µm] ≤ 48<br>M2 Cytoplasm Width [µm] ≤ 40<br>M2 Cytoplasm Ratio Length to Width 0≤X≤0.37<br><br>**Output Population:** M2 macrophage	
	Population: M2 Macrophages<br>Method: Filter by Property<br>Parameter: Number of Leishmania per cell >0<br><br>**Output Population:** infected M2 macrophage	
	**Calculate Readout Values**	
Define Results	Method: List of outputs<br>Populations: Macrophages<br>M1 macrophages<br>M2 macrophages<br>Total *Leishmania*<br>Mean of *Leishmania* per cell<br>Infected macrophages<br>Double infected macrophages<br>Massive infected macrophages<br>Infected M1 macrophages<br>Infected M2 Macrophages<br>Method: Formula Output: a/b<br>Variable a: *Leishmania*<br>Variable b: macrophages<br>Output: *Leishmania* per macrophages<br>Variable a: Leishmania<br>Variable b: infected macrophages<br>Output: *Leishmania* per infected macrophages<br>Method: Formula Output a/b*100<br>Variable a: infected macrophages<br>Variable b: macrophages<br>Output: Percent infected macrophages	Calculation of readouts to quantify biological effects visible in fluorescence pictures

ROI= Region of interest
